# Spectral consensus strategy for accurate reconstruction of large biological networks

**DOI:** 10.1186/s12859-016-1308-y

**Published:** 2016-12-13

**Authors:** Séverine Affeldt, Nataliya Sokolovska, Edi Prifti, Jean-Daniel Zucker

**Affiliations:** 1 Integromics, Institute of Cardiometabolism and Nutrition, ICAN, Assistance Publique Hôpitaux de Paris, Pitié-Salpêtrière Hospital, Paris, 75013 France; 2 Sorbonne Universités, UPMC University Paris 6, UMR S U1166 NutriOmics Team, Paris, 75013 France; 3 UMR S U1166 Nutriomics Team, INSERM, Paris, 75013 France; 4 IRD, UMI 209, UMMISCO, IRD France Nord, Bondy, F-93143 France

**Keywords:** Network reconstruction, Community-based method, Spectral theory, High-dimensional data, Microbiota

## Abstract

**Background:**

The last decades witnessed an explosion of large-scale biological datasets whose analyses require the continuous development of innovative algorithms. Many of these high-dimensional datasets are related to large biological networks with few or no experimentally proven interactions. A striking example lies in the recent gut bacterial studies that provided researchers with a plethora of information sources. Despite a deeper knowledge of microbiome composition, inferring bacterial interactions remains a critical step that encounters significant issues, due in particular to high-dimensional settings, unknown gut bacterial taxa and unavoidable noise in sparse datasets. Such data type make any a priori choice of a learning method particularly difficult and urge the need for the development of new scalable approaches.

**Results:**

We propose a consensus method based on spectral decomposition, named *Spectral Consensus Strategy*, to reconstruct large networks from high-dimensional datasets. This novel unsupervised approach can be applied to a broad range of biological networks and the associated spectral framework provides scalability to diverse reconstruction methods. The results obtained on benchmark datasets demonstrate the interest of our approach for high-dimensional cases. As a suitable example, we considered the human gut microbiome co-presence network. For this application, our method successfully retrieves biologically relevant relationships and gives new insights into the topology of this complex ecosystem.

**Conclusions:**

The *Spectral Consensus Strategy* improves prediction precision and allows scalability of various reconstruction methods to large networks. The integration of multiple reconstruction algorithms turns our approach into a robust learning method. All together, this strategy increases the confidence of predicted interactions from high-dimensional datasets without demanding computations.

**Electronic supplementary material:**

The online version of this article (doi:10.1186/s12859-016-1308-y) contains supplementary material, which is available to authorized users.

## Background

Discovering complex interactions is a long-standing problem which led over the past years to the development of many network reconstruction methods that exhibit competitive results on various types of data. As successfully demonstrated, networks are invaluable tools to comprehensively relate biological variables [1–3] and possibly gain insights into their direct causal relationships [4]. Interestingly, recent studies have shown that the available approaches would not generally perform optimally across all dataset types and the integration of diverse inference methods can provide an improved robust performance [5–8]. However, several well-known and widely used algorithms cannot directly process high-dimensional data or actually perform better on small networks. Bringing these methods within a lower dimensional space would enable researchers to fully benefit from their strengths under high-dimensional settings, and more interestingly, to integrate their outcome in community-based predictions.

We propose a *consensus* approach, named *Spectral Consensus Strategy* (SCS), to reconstruct complex biological networks from high-dimensional datasets. This method provides scalability to various reconstruction methods and can be applied to a broad range of complex biological networks. Our approach unfolds in three parts. First, it relies on a spectral framework to identify sets of significantly related variables. Specifically, the subset selection uses the magnitude of the normalized Laplacian eigenvector elements. These subsets are then considered in a second phase for multiple parallel *local* reconstructions from which global effects are inferred. By enabling each reconstruction method to locally *avoid* high-dimensional settings, this second phase improves individual prediction accuracy and scalability. In the last phase, the individual reconstructions that benefited from the spectral embedding are integrated in a consensus network.

All together, this strategy provides robust and accurate reconstructions from high-dimensional observational data for which no suitable learning approach is known beforehand, as for instance frequently encountered in metagenomics. To our knowledge, our contribution is the first attempt to introduce a consensus network reconstruction approach based on a spectral framework.

### Network reconstruction background

Generally speaking, network learning algorithms can be divided into two categories: *constraint-based* and *score-based* approaches. The constraint-based methods ascertain (conditional) independence relationships from statistical tests [9, 10] to learn structural constraints in causal graphs. These approaches are highly efficient on sparse networks and are guaranteed to learn the Markov equivalent class of the underlying graphical model if the *exact* list of conditional independence relationships is given. However, constraint-based methods have also proved to be very sensitive to sampling noise from finite datasets. Alternatively, score-based methods identify the model that best fits the data through the maximization of a score function over the space of (ideally all) possible Bayesian networks [11, 12]. To learn the networks in reasonable time, the search procedure usually follows a heuristic algorithm that identifies a local optimum. More recently, several *mutual information-based* approaches have been proposed to infer direct relationships from noisy observational datasets containing few samples [1, 2]. Nevertheless, as demonstrated by the growing number of *hybrid* approaches [4, 13–15], the wide range of high-dimensional data is still challenging state-of-the-art methods, both in terms of accuracy, or time and memory consumption.

### Spectral methods background

Spectral theory has provided a number of approaches to uncover dataset structure. A well-known result is the ability to optimally bi-partition a graph based on the second eigenvector of the normalized Laplacian matrix, also known as *algebraic connectivity* or *Fiedler vector* [16, 17]. Following this idea, recursive *two-way* cut methods [18–20] that rely solely on the second eigenvector, and *k-way* cut approaches [21–26] that are based on truncated eigenvector basis, have been successfully applied to dimensionality reduction or clustering problems. Specifically, the truncated eigenvector basis provides a new representation that amplifies the similarity between closely related variables while reducing the affinity of unrelated variables [26–29]. Many biological systems are usually composed of overlapping sub-units that involve functionally related features, such as found in metabolic or gene regulatory networks. Hence, learning large biological networks from multiple local reconstructions appears to be a reasonable procedure as much as it follows the natural dataset structure. Spectral methods hold great potential for guiding learning algorithms that perform better on small graphs towards improving inference of large networks.

### Consensus reconstruction approaches

The idea of consensus or *ensemble* learning is recently gaining interest in the field. An example is given in [30] where the yeast metabolic network was reconstructed based on a complex consensus procedure that involved a number of statistical methods and an important amount of prior knowledge. As previously demonstrated [31], consensus approaches can be efficiently exploited to reconstruct Bayesian networks and provide robust models from biological data. A consensus method that mainly rely on significance tests is proposed in [32] to learn dependencies between gene regulatory factors in the human frontal lobe, resulting in a high-confidence model. The community structure in complex networks can also be revealed by consensus clustering as reported in [33], where a stable partitioning approach based on several stochastic method results is proposed. Marbach et al. [5] motivates the development of consensus methods by demonstrating the benefits of combining complementary inference approaches. Specifically, they have evaluated the performance of diverse learning algorithms and shown that their combination performs robustly across various datasets while providing as good or better results than individual methods.

### The complex gut microbiome system

The human gut hosts a high density of commensal bacteria whose collective genome, also known as *metagenome*, exceeds more than a hundred times the size of the human genome [34]. This rich ecosystem provides the host with vital functions that affect nutritional efficiency and overall health [35, 36]. Over the past few years, the role of gut microbiota in human health has received unprecedented attention [37]. In particular, several chronic diseases such as obesity [38, 39], inflammatory bowel disease [40, 41], liver cirrhosis [42, 43], type-I [44], and type-II diabetes [45, 46] have been associated with gut microbiota. For a long time, the composition of human gut microbial ecosystem was unknown, especially due to the large number of non-cultivable species. The recent availability of metagenomic data along with different binning techniques allows now to obtain a better picture of the taxonomical groups that inhabit the gut microbiome [47]. These species are organized in complex ecological networks and can be involved in different types of interactions such as competition or mutualism [48]. Yet, mapping these relationships with high confidence remains a complicated task for multiple reasons. First of all, as many species are usually absent from one sample to another, metagenomic datasets are very sparse. This sparsity adds on technical artifacts inherent to the obligate multi-step data processing. Hence, metagenomic data are challenging available reconstruction methods, which may individually yield different topologies for the same set of observations.

## Methods

**Fig. 1:**
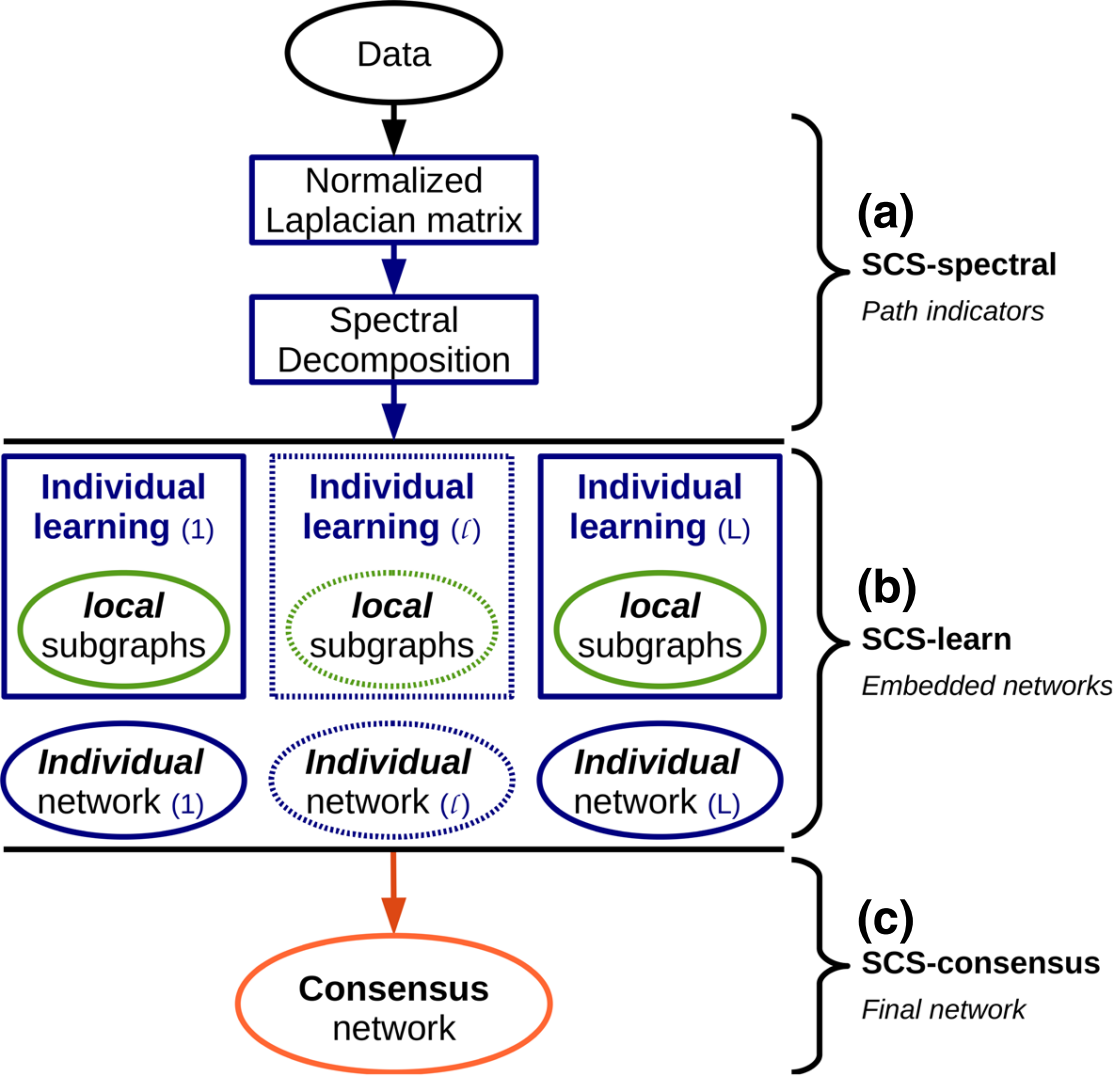
Overview of the Spectral Consensus Strategy (SCS). The SCS method unfolds in three parts. **a** The SCS-*spectral* phase identifies sets of *path-related* variables based on the magnitude of the graph Laplacian eigenvector elements. **b** The SCS-*learn* phase performs multiple parallel *local* reconstructions using different learning methods. **c** The SCS-*consensus* phase provides a consensus network built on the individual outcomes from the SCS-*learn* step

We propose a simple yet highly efficient method called *Spectral Consensus Strategy* (SCS) that simultaneously embeds multiple discovery algorithms within a spectral framework for the reconstruction of large graphical model. The strength of the SCS method hinges on two key points that are (i) the accuracy improvement of each individual learning algorithm and (ii) the combination of predictions from complementary reconstruction methods. Specifically, sets of *path-related* variables are first identified based on the magnitude of the graph Laplacian eigenvector elements (Fig. 1,a), then multiple parallel local reconstructions are performed using different learning methods (Fig. 1,b) and lastly a consensus network is built on the previous multiple outcomes (Fig. 1,c).

In the following, we provide theoretical support to the uncovering of connected variable subsets from the first phase of the SCS approach (SCS-*spectral* step, Fig. 1,a). In particular, we demonstrate that subsets of *path-related vertices* can be directly retrieved from the magnitude and sign of individual eigenvector elements. These subsets, which correspond to possibly overlapping dense subgraphs, are given as input to the second phase of the SCS approach (SCS-*learn* step, Fig. 1,b). We finally detail the whole *Spectral Consensus Strategy*.

### Normalized Laplacian eigenvectors

We consider the *random-walk* normalized Laplacian matrix *L*_*rw*_ as it entails the random walk dynamics from one vertex to another in the corresponding graph $\mathcal {G}$. This matrix is defined as *L*_*rw*_=*I*−*D* ^−1^ *W*, where *I* is the identity matrix, *W*=(*w*_*ij*_) is a weight matrix over all pairs of variables and *D* the diagonal degree matrix with $d_{ii}=\sum _{j}w_{ij}$.

#### Community membership indicators

As already established [49], the null eigenvalues of the graph Laplacian matrices are associated with the number of *connected components*. A subset of vertices *A*_*k*_⊂*V* is a connected component if (i) all intermediate points that lie on a path between two vertices of *A*_*k*_ also belong to *A*_*k*_ and (ii) there is no connection between the vertices of *A*_*k*_ and its complementary subset $\overline {A_{k}}$ (Additional file 1: Proposition 1). Interestingly, for the case of finding *k*>2 clusters, the first *k* eigenvectors of the normalized Laplacian matrix *L*_*rw*_ minimize the *normalized cut* (*N* *C* *u* *t*) criterion of the relaxed problem [18, 50],

1 $$ \mathit{Ncut}(A_{1},\ldots,A_{k})=\frac{1}{2} \sum_{i=1}^{k} \frac{W(A_{i},\overline{A_{i}})}{vol(A_{i})}  $$

where $W(A,B)=\sum _{\substack {i \in A \\ j \in B}} \omega _{ij}$, and $vol(A_{i})=\sum _{j \in A_{i}} d_{j}$.

In a nutshell, the solution of the relaxed *N* *c* *u* *t* minimization problem consists of the orthonormal matrix $H \in \mathbb {R}^{p \times k}$ whose columns are the first *k* eigenvectors of the normalized Laplacian eigenvector matrix *U*, associated with the first *k* smallest eigenvalues.

When the between-cluster similarity is exactly 0, these eigenvectors are the indicator vectors {*h*_*j*_}_*j*∈[1,*k*]_ ($h_{j} \in \mathbb {R}^{p}$ and ${h_{j}^{i}}=1$ if *x*_*i*_∈*A*_*j*_, otherwise 0) of the *k* connected components [50]. In practice, the distribution of the data points in distinct clusters is hardly encountered, and one should expect the between-cluster similarity to be greater than 0. Yet, under *nearly ideal* conditions the eigenvectors are still close to the indicator vectors, and the elements magnitude and sign of each eigenvector contain information on vertices membership *strength* [18, 50, 51].

#### Path-related vertices subsets

Beyond the membership indication, the Laplacian matrix eigenvector elements also convey path-relationship information. In the following we assume that *v*_*k*_ is the *k*-th eigenvector of the normalized Laplacian matrix associated with the connected component *A*_*k*_. Under ideal conditions, *x*_*i*_∈*A*_*k*_⇒*v*_*k*_(*i*)=1, otherwise *v*_*k*_(*i*)=0 [50]. In addition, we demonstrate that similar elements of a given eigenvector (|*v*_*k*_(*i*)−*v*_*k*_(*j*)|=0) indicate path-related variables (*x*_*j*_ is path connected with *x*_*i*_) based on the Rayleigh quotient [52] (Additional file 1: Proposition 2).

For the case of a *connected* graph $\mathcal {G}$ (i.e. there is a path between any pair of variables in $\mathcal {G}$) Fiedler’s Nodal Domain theorem (Additional file 1: Theorem 1) indicates that while *x*_*i*_ and *x*_*j*_ belong to different clusters *A* and *B*, |*v*_*k*_(*i*)−*v*_*k*_(*j*)|<*ε* can be found. However, if there exists a subset of vertices *S* at a distance less than a *step* *ρ*≥2 from *A* that separates *A* and *B*, then *v*_*k*_ is such that [53]

$$ \left\{\begin{array}{ll} & \text{if}~i \in A, \, \text{then}~v_{k}(i) = 1,\\ & \text{if}~i \in B, \, \text{then}~v_{k}(i) = -1,\\ & \text{if}~i \in S, \, \text{then}~-1 + 2/\rho \leq v_{k}(i) \leq 1 - 2/\rho, \\ & \text{if}~i,j \text{ are adjacent then}~|v_{k}(i)-v_{k}(j)| \leq 2/\rho. \end{array}\right.  $$

Taking *ρ*=2 we obtain the case which is commonly used for separators. Hence, |*v*_*k*_(*i*)−*v*_*k*_(*j*)| is a measure of the distance between the vertices *i* and *j* reflecting the *cluster assumption* which stipulates that close data points are expected to lie within the same cluster (Additional file 1: Proposition 3).

In summary, under ideal conditions, the first *k* eigenvectors of the normalized Laplacian matrix provide indicator vectors of the *k* connected components. In practice, the magnitude and sign of the eigenvector elements contain information on vertex membership *strength* to the corresponding component (Additional file 1: Proposition 1). Furthermore, *path-connected* variables have similar eigenvector elements (Additional file 1: Proposition 2), that are distinct from the element of vertices belonging to a different component (Additional file 1: Proposition 3). Thus, subsets of nodes that correspond to large positive or negative eigenvector elements (retrieved in the SCS-*spectral* step) correspond to dense subgraphs (to be reconstructed in the SCS-*learn* step). These subgraphs associated to large eigenvector elements can be redundantly found in the first eigenvectors [54]. However, higher eigenvectors can also be used to identify different subsets of connected nodes, as observed in the context of anomalous graph detection [55].

### The spectral consensus strategy

This section details the three steps of the SCS approach and provides the algorithms associated with each phase (Fig. 1).

#### (a) SCS-spectral, identifying graph sub-paths

The first phase of the SCS approach, called SCS-*spectral*, identifies subsets of vertices that are at a small *walk* distance from each other within the graph $\mathcal {G}$ (Fig. 1,a). This information is conveyed by the magnitude of the Laplacian eigenvector elements [51].

In the following, the input data matrix is $\mathbb {R}^{n \times p}$ with *n* the number of observations and *p* the number of variables. In Algorithm 1, the eigenvectors of the normalized Laplacian matrix *L*_*rw*_ are computed to identify vertices that lie on common sub-paths (Algorithm 1, lines 4−5).



In our consensus approach, we choose the mutual information to model vertex similarity as it provides a general measure of relationship between variables [56, 57]. Moreover, previous studies have shown that information theoretic measures are well suited to study high-dimensional biological data [58–60], which was one of our objectives when designing the SCS approach.

#### (b) SCS-learn, high-dimensional spectral embedding

The second phase of our approach, called SCS-*learn*, relies on the sign and magnitude of the first *k* eigenvector elements to reconstruct possibly overlapping sub-graphs that involve path connected vertices (Fig. 1,b). Specifically, each eigenvector *v*_*k*_ is associated with two sub-graphs, $\mathcal {G}_{v_{k}}^{m,-}$ and $\mathcal {G}_{v_{k}}^{m,+}$, that relate the *m* data points corresponding to either the most *negative* or the most *positive* eigenvector elements (Algorithm 2, line 7).



For clustering purposes, the subspace spanned by the first *k* eigenvectors would normally be preferred to their individual interpretation [28]. However the SCS-*learn* step does not aim at partitioning the variables, but rather to learn the whole underlying network based on overlapping sub-graphs. In particular, the non high-dimensional settings (*m*≪*n*) obtained for each local reconstruction $\mathcal {G}_{v_{k}}^{m,\scriptscriptstyle {+/-}}$ restrict the number of *false positive* edges. Alternatively, the overlaps between selected subsets of *m* variables limit the number of *false negative* interactions. At the end of this phase, the edges eventually retained in each individual network $\mathcal {G}_{l}$ are those that were learned every time a sub-graph $\mathcal {G}_{v_{k}}^{m,\scriptscriptstyle {+/-}}$ involved the corresponding pair of vertices (Algorithm 2, lines 17−18). Lastly, whenever the input reconstruction method $\mathcal {R}_{l}$ provides orientations, a *majority rule* is applied to set the final orientation or resolve possible conflicts over all the inferred orientations for two adjacent vertices (Algorithm 2, line 19). If no majority can be achieved, the edge is set undirected.

#### (c) SCS-consensus, final network



In this last phase, called SCS-*consensus*, networks inferred by individually embedded reconstruction methods are combined (Fig. 1,c). Specifically, for each learning approach $\mathcal {R}_{l}$, we rank the predicted edges by decreasing strength or confidence (Algorithm 3, lines 4−6). Then, following the integration procedure proposed in [5], an average is computed to provide a consensus rank for the (*x*_*i*_,*x*_*j*_) edge in the final graph $\mathcal {G}$ (Algorithm 3, line 8). If an individual reconstruction method gives no edge between (*x*_*i*_,*x*_*j*_), the pair receives the worst possible rank for this method, i.e. $rank^{x_{i}x_{j}}_{\mathcal {G}_{l}}=1$. A weighted average over the (sub)set $\{\mathcal {R}\}_{L'}$ of learning approaches that predicted orientations is also computed, giving greater weight to upper rank edge orientations (Algorithm 3, lines 9−12). Lastly, only the *e*_*max*_ most significant edges are retained in the consensus network (Algorithm 3, line 15).

## Results

**Fig. 2:**
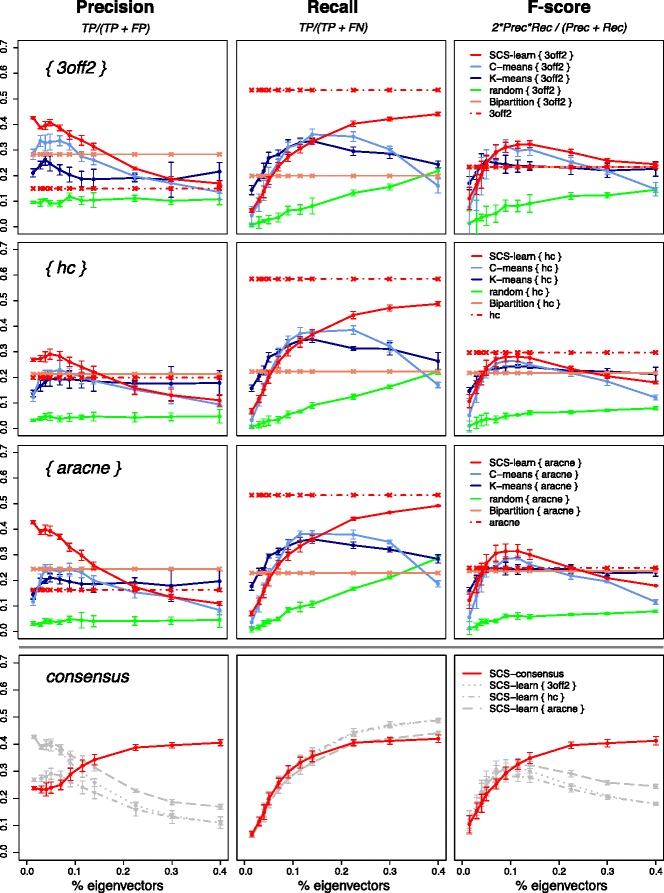
SCS-learn and SCS-consensus evaluations for ANDES benchmark network [223 nodes, 338 edges, 〈*k*〉=3.03]. *Precision*, *Recall* and *F-score* results for an increasing proportion of eigenvectors (up to 40 *%*), subgraphs of 12 nodes (5 *%* variables) and 150 samples. Scores take misorientations into account. Each point is an average over 5 datasets (results for different subgraph and dataset sizes follow a similar trend, see Additional file 1). (SCS-learn, top three rows) Three learning algorithms are embedded to reconstruct a network from subgraphs whose vertices are selected from the magnitude of eigenvector elements (SCS-*learn*, red solid line), spectral fuzzy C-means partitioning (*light blue* solid line), spectral K-means clustering (*dark blue* solid line), random subsets (*green* solid line) and recursive bi-partitioning (salmon solid line). Results are compared to scores obtained without spectral or partitioning embedding (*red* dashed line). (SCS-consensus, bottom row) The SCS-*learn* reconstructions are combined in a consensus network (*red* solid line) and compared with individual SCS-*learn* outcomes (*gray* dashed lines). Scores are computed from the top 338 consensus edges (results for different number of consensus edges follow a similar trend, see Additional file 1)

The SCS approach embeds multiple reconstruction methods in a spectral framework to learn possibly oriented interactions from high-dimensional data by (i) combining the edges discovered from overlapping sub-graphs (Fig. 1, SCS-*learn*, (b)) and (ii) computing a consensus network (Fig. 1, SCS-*consensus*, (c)). In the following, the reconstructed networks are evaluated for an increasing proportion of eigenvectors (Fig. 2, horizontal axis). Results are discussed in terms of *Precision* (*T* *P*/(*F* *P*+*T* *P*)), *Recall* (*T* *P*/(*T* *P*+*F* *N*)) and *F-score* (2×*P* *r* *e* *c*×*R* *e* *c*/(*P* *r* *e* *c*+*R* *e* *c*)) (*F* *N*,*T* *P*,*F* *P*; *false negative*, *true positive* and *false positive* edges resp.). In particular, falsely oriented *T* *P* edges are considered as *F* *P*. For these evaluations, a benchmark network of 223 nodes and 338 edges has been considered (ANDES benchmark [61, 62]). This choice was in particular motivated by the fact that each variable of the ANDES benchmark network has exactly two categories, as encountered for metagenomics co-presence or presence-absence data. Besides, the 223 variables of this network enable us to reproduce high-dimensional conditions while evaluating the SCS results against reconstruction performed by each learning approach without the SCS embedding. We also considered a larger benchmark network composed of 1, 041 nodes and 1, 397 edges, MUNIN [63], and provide the corresponding results in (Additional file 1: Figures S3 and S9). We randomly sampled 5 datasets of sizes 150 and 200 to perform the experiments under high-dimensional conditions for ANDES, and 5 datasets of size 935 for MUNIN. The embedded reconstruction methods are ARACNE [1], a mutual information-based approach, 3off2 [4], a hybrid method that combines constraint-based and scoring approaches based on multivariate information measures, and a hill-climbing algorithm using the Bayesian Dirichlet equivalent score. We also considered a random classifier in our SCS-*spectral* and SCS-*learn* step evaluations (Additional file 1: Figures S4).

### SCS-learn network evaluations

As previously established [5], adding high quality reconstruction methods to a consensus approach significantly improves consensus predictions. We have thus evaluated the accuracy improvement achieved in the SCS-*learn* phase that relies on the SCS-*spectral* step. Specifically, we have compared reconstructions obtained from variable subsets selected with the element magnitude of the first *k* eigenvectors to networks learnt based on variable subsets derived from different partitioning or clustering methods. Alternative subset selections are provided by spectral fuzzy C-means partitioning, spectral K-means clustering and recursive bi-partitioning. Random subset selection is also considered as a mere comparison.

Evaluations of embedded network reconstructions from subgraphs of *m*=12 nodes using *n*=150 samples (results for different subgraph and dataset sizes follow a similar trend, see Additional file 1) for the ANDES benchmark are given in Fig. 2 (top three rows). Reconstructions obtained from randomly sampled subsets exhibit a poor *Precision* (green solid line). This highlights that guided local reconstructions improve prediction accuracy. Networks reconstructed from subgraphs that rely on spectral K-means (darkblue solid line) or spectral fuzzy C-means (lightblue solid line) subsets do not provide better *Precision* than the SCS-*learn* method (red solid line) up to 30 eigenvectors (14 *%* of the total number). Although bipartition of the variables (salmon solid line) allows for better *Precision* than the random or spectral clustering, it is still largely outperformed by the SCS-*learn* phase.

This high *Precision* is at the slight expense of the *Recall* (Fig. 2, middle column), although it still outperforms the bi-partitioning approach and performs almost as better as clustering-based reconstructions. It is worth noting that reconstructions obtained with the SCS-*learn* step are consistent with Proposition 2 and 3. In particular, Fig. 2 shows an increase of the *Recall* as the number of eigenvectors grows (middle column, red solid line) as well as a higher *Precision* with the first eigenvectors (left column, red solid line). This is in line with a progressive discovery of the true underlying network and further show that non principal eigenvectors, although less informative than the first eigenvectors, carry relevant information on connected vertices. This can also be observed, to a lesser extent, when a random classifier is embedded in the SCS-*learn* step (Additional file 1: Figure S4). Interestingly, the *Recall* of networks based on spectral clustering partitions decreases when too many eigenvectors are considered (Fig. 2, middle column, lightblue and darkblue solid lines). As already established [26–29], truncated eigenvector basis are expected to emphasize variable similarities and thus, should indicate relevant variable subsets. Yet, due to the approximation error from the real valued solution, non principal eigenvectors are unreliable and worsen variable partitioning. Consequently, connected vertices may be assigned to distinct clusters as the number of eigenvector grows, leading to local reconstructions with a low *Recall*.

All together, the association of the SCS-*spectral* and SCS-*learn* steps leads to higher *F-score* results (Fig. 2, right column; Additional file 1: Figure S3, left column) as compared to reconstructions obtained with various partitioning approaches. This improvement is achieved from a relatively small number of eigenvectors (5 *%* of the total number), thus enabling a good trade-off between reconstruction quality and the number of required subgraphs. Lastly, the ANDES benchmark network was considered as its size allows for a direct reconstruction by each learning method. Results provided in Fig. 2 (dashed red line) show that SCS-*learn* performs better than, or as well as, reconstruction methods alone.

### SCS-consensus network evaluations

Evaluations of consensus networks reconstructed from embedded learning approaches based on subgraphs of *m*=12 nodes and using *n*=150 samples are given in Fig. 2 (bottom row). The ANDES benchmark network having 338 edges, scores for the consensus outcome are given based on the 338 first ranked edges (results for different number of edges follow a similar trend, see Additional file 1). The consensus *Precision* scores (Fig. 2, bottom left, red solid line) clearly outperform the individually embedded learning approaches (gray dashed lines) as the proportion of eigenvector grows. Similar results are observed for the MUNIN benchmark network (Additional file 1: Figure S9).

Interestingly, these results emphasize the complementarity of the different reconstruction methods, as already demonstrated [5]. In particular, it has been shown that ARACNE and other mutual information reconstruction methods detect more easily feedfoward loop (*A*→*B*→*C* and *A*→*C*) and fan-in (*A*→*C* and *B*→*C*) patterns. Conversely, cascade (*A*→*B*→*C* and (*A*,*B*) not adjacent) and fan-out (*A*→*C* and *A*→*B*) patterns are more easily inferred by Bayesian learning approaches [5].

All together, the SCS-*consensus* phase provides high *F-score* network reconstructions (Fig. 2, bottom right, red solid line) for a reasonable number of eigenvectors (proportion ≥11.5 *%*). The SCS-*consensus* predictions also exhibit high *F-scores* when considering variable subsets of larger sizes in the SCS-*learn* phase (Additional file 1: Figures S7–S9).

### Reconstruction of microbial ecosystems

We applied the SCS method to a complex biological dataset generated by high-throughput sequencing of gut microbiome samples from 663 patients recruited in the MetaHIT project (Metagenomics of the Human Intestinal Tract). The nearly 4 million genes whose abundance was measured using quantitative metagenomics were binned to generate representative variables based on their mean co-abundance as introduced by Nielsen et al. [47]. These co-abundance groups (CAG) can be either classified as *genomic units* (GU) for small groups (between 3 and 700 genes) or *metagenomic species* (MGS) for larger groups (more than 700 genes). The authors produced a first reconstruction of the gut microbial ecosystem based on Fisher’s exact test between pairs of CAGs.

**Fig. 3:**
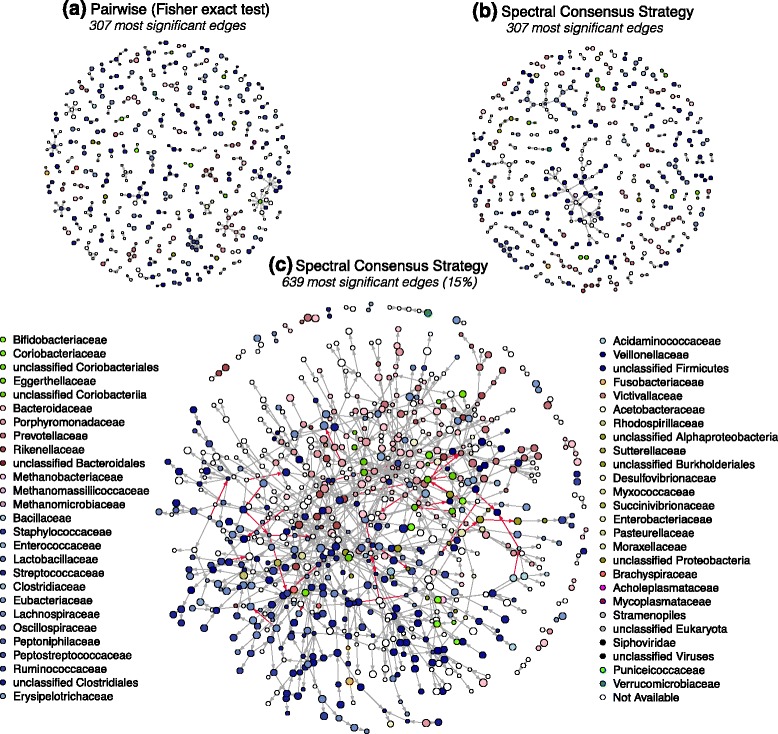
Microbial co-presence ecosystem. Microbial ecosystem reconstructed with the pairwise Fisher’s exact test [47] (**a**) and the SCS approach (**b,c**). Data for 2, 101 co-abundant groups (CAGs) and *n*=663 patients recruited in the MetaHIT project were used. Edges depict co-presence (*gray edges*) or absence-presence (*red edges*) relationships. **a** Gut microbial ecosystem based on Fisher’s exact test between pairs of CAGs [47] (307 edges between 445 CAGs of at least 50 genes). **b** The same number of top-ranked edges (307) obtained with the SCS approach which involve 443 CAGs of at least 50 genes. **c** The 15 *%* most significant edges obtained with the SCS approach (654 nodes and 639 edges)

In our study we used this extensively annotated dataset where information on phylogenetic classification and gene assembly is also available. Here we focused on *p*=2, 101 CAGs with more than 50 genes as already proposed in [64]. Figure 3 a represents 307 co-presence relationships (edges) between these 2, 101 CAGs (vertices) with at least one connection (leading to a subset of 445) as already provided by Nielsen et al. [47]. The number of genes composing a CAG is proportional to the vertex size. CAGs from the same phylum have similar color hues that are specified at the family level of their phylogenetic classification (e.g. *Firmicutes* are given in a range of blue and *Bacteroides* in a range of pink).

The SCS approach which embeds three reconstruction methods (ARACNE, 3off2, hill-climbing) inferred a consensus network of 6, 389 edges from the above-mentioned dataset. To compare our results with the pairwise network reconstructed by Nielsen et al. [47], we selected the same number (307) of top-ranked SCS edges which represent approximately 5 *%* of the consensus interactions. This network composed of 443 nodes yields more complex sub-structural patterns as illustrated in Fig. 3 b. When comparing networks (A) and (B), only 111 out of the 307 edges (36 *%*) inferred by Fisher’s exact test are also predicted by the SCS method. Interestingly, 105 of these common edges (95 *%*) have genetic elements that share same assembly contigs, bringing strong biological evidence for these predicted relationships. Conversely, out of the remaining 196 edges solely inferred by Fisher’s exact test, a significantly smaller number (121, 62 *%*) have genetic elements that share same assembly contigs (*p*<8×10^−10^, *χ* ^2^). Complementary evaluations for different number of common edges (from 55 to 146 edges) follow the same trend (Additional file 1: Table S4 and Additional file 1: Figure S9). We hypothesize that a non negligible number of edges inferred by pairwise reconstruction techniques may correspond to indirect relationships.

We explored the topology of the SCS consensus gut microbial ecosystem at different most significant edges threshold (*e*_*max*_) and illustrate the network at 15 *%* in Fig. 3 c (654 vertices and 639 edges). The modular structure of this network is highlighted by tightly related vertices sharing similar colors. This indicates that species of the same family or phylum are mostly co-present as previously discussed [43]. This can be explained by the fact that closely related species have similar genetic background adapted for the same environmental niche. Of interest is also the fact that small CAGs (GU) are strongly linked with large CAGs (MGS) having the same phylogenetic annotations as depicted in Fig. 3(a & b) and previously described [47]. The SCS microbial network also includes consensus directed edges computed from the orientations of the embedded 3off2 and hill-climbing algorithms. Gray oriented edges (*A*→*B*) indicate *ordered* co-presence relationships (i.e. the presence of *A* species is expected whenever *B* is found). Conversely, red oriented edges provide presence-absence information.

**Fig. 4:**
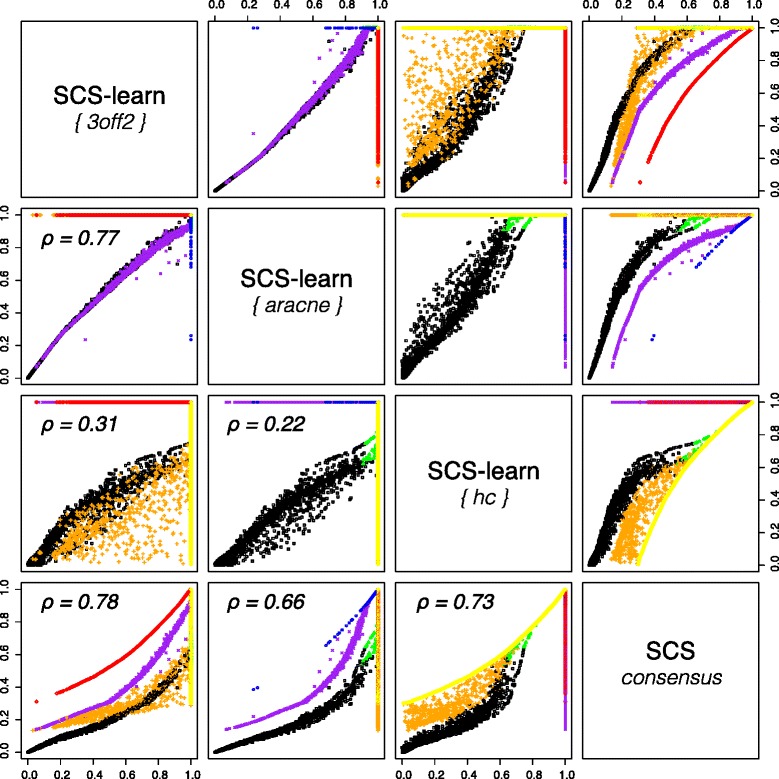
Edge rank correlations between SCS-learn and SCS-consensus outcomes for human gut microbial ecosystem. 6, 389 edges were predicted from a dataset of 663 observations and 2, 101 CAGs (MetaHIT project [47]). Rank of edges predicted by only one embedded learning method are given in blue (ARACNE, 159 edges), red (3off2, 498 edges) and yellow (hill-climbing, 2, 889 edges). Rank of edges predicted by two individual learning methods are given in green (ARACNE & hill-climbing, 31), orange (3off2 & hill-climbing, 573 edges) and purple (3off2 & ARACNE, 720 edges). Rank of edges predicted by all individual methods are given in black (1, 519 edges)

We further analysed the SCS microbial network by considering the edge rank correlations between individual reconstructions and the consensus result (Fig. 4). The 3off2 and the ARACNE algorithms have a strong correlation (Fig. 4, *ρ*=0.77), as it could be expected for approaches that rely on similar metrics. Conversely, the edge ranks between 3off2 or ARACNE and hill-climbing heuristic exhibit weak correlation coefficients (Fig. 4, *ρ*=0.31 and *ρ*=0.22 resp.). The slightly higher correlation between 3off2 and hill-climbing approaches may be related to the fact that 3off2 is a hybrid approach that is also score-based. All together, these results demonstrate the complementarity of the individual approaches from which the human gut microbial consensus predictions can benefit.

## Discussion

In this paper, we propose a consensus network learning approach called *Spectral Consensus Strategy* which is based on spectral decomposition. Our method proceeds in three steps, namely SCS-*spectral*, SCS-*learn* and SCS-*consensus*. The first and second phases enable any reconstruction method to learn a possibly oriented network under high-dimensional settings. In addition to accuracy improvement of each reconstruction method, the spectral framework on which the SCS approach relies, also supports fast processing of high-dimensional datasets. The last phase combines the outcome of each reconstruction method to provide consensus predictions.

This strategy, as well as being accurate, scales up extremely well. Specifically, as the SCS-*learn* step processes in parallel local reconstructions related to the first *k* eigenvectors (Algorithm 2, lines 5−15), it is the time complexity of the reconstruction methods that mainly impedes the whole running time. The SCS framework itself does not add any demanding computations. In particular, the running time for each individual reconstruction method embedded in the SCS-*learn* phase grows with the number of variables *p* as $\mathcal {O}(p\log {p})$ (Algorithm ??, line 6). Furthermore, all reconstruction methods can simultaneously learn the network within the second phase. As an example, gut microbiota consensus reconstruction (2, 101 variables, 663 samples, Fig. 3 c) required 43 seconds to reconstruct all subgraphs (*m*=40 vertices, 63 eigenvectors) needed for the $\mathcal {G}_{l}$ individual networks, and 52 seconds to build the consensus outcome $\mathcal {G}$ using 40 CPUs. Besides, the early step of the SCS-*spectral* phase which involves the computation of the mutual information matrix (Algorithm ??, line 3) and the last step of the SCS-*learn* phase which is dedicated to the assembling of local reconstructions (Algorithm ??, lines 17−19), can be efficiently optimised and implemented [65, 66]. All together, the SCS approach could efficiently reconstruct the microbiome ecosystem, while the hill-climbing algorithm alone did not converge in 48 hours (see Additional file 1: Section 4, for detailed evaluations). These results highlight the ability of our method to improve the scalability of the embedded learning approaches.

The subgraph size *m* for the SCS-*learn* phase influences the quality of individual reconstructions ($\mathcal {G}_{l}$ graphs). Specifically, too small subgraphs lead to low *Recall* and very high *Precision*, while conversely too large subgraphs (even still under non high-dimensional conditions) increase the *Recall* at the expense of the *Precision*, both cases impeding the *F-score* results (Additional file 1: Figures S1–S3). Yet, predictions output by the SCS-*learn* step remain better than predictions derived from classical clustering and partitioning approaches for various sizes *m*. Interestingly, although the parameter *m* significantly impacts individual reconstructions, it only slightly impedes the consensus *F-score*. In particular, larger subgraphs still provide a consensus network of good quality from high-dimensional dataset (Additional file 1: Figures S7–S9). Similarly, the eigenvector proportion influences individual reconstructions $\mathcal {G}_{l}$ as too many eigenvectors lead to lower *Precision* and higher *Recall*. Yet, the consensus network based on the first *e*_*max*_ most significant edges achieves good and stable quality as the number of eigenvectors grows (Additional file 1: Figures S7–S9).

To define the minimal number of eigenvectors that would bring sufficient amount of information for a good consensus reconstruction, we designed a heuristic approach based on the decreasing interval between successive eigenvalues. For classical clustering approaches, the *eigengap* heuristic has been proposed to define the most suitable cluster number. This eigengap heuristic method is related to the fact that under ideal conditions, *k* distinct connected components are associated to the first *k* null eigenvalues and thus, a gap can be found between *λ*_*i*≤*k*_=0 and *λ*_*k*+1_>0. In practice, the eigengap heuristic sets the number *k* such that *λ*_*i*≤*k*_ are small but *λ*_*k*+1_ is relatively large. The SCS approach objective is not to partition variables but rather to reconstruct a consensus network from overlapping subgraphs, using as much as possible of the information conveyed by each eigenvector. As shown from the counts and cumulative counts of *true positive* interactions for the ANDES benchmark network (Additional file 1: Figure S5), although most of the *true positive* interactions are retrieved from the first eigenvectors, non principal eigenvectors also conveyed relevant information on connected vertices. Hence, we consider the first *k* eigenvectors for which the successive eigenvalues are dissimilar enough as being the best number of eigenvectors to be used for the SCS consensus reconstruction. As an example, our heuristic method evaluated at 30 (14 *%*) the most suitable number of eigenvectors for the ANDES benchmark network. This number approximately corresponds to the number of eigenvectors from which the consensus network achieves better *F-score* results than networks obtained from individually embedded methods (Fig. 2).

The SCS approach is mainly designed to reconstruct large unknown biological networks, thus no weights have been assigned to individual reconstruction methods. However, if any prior knowledge is available on the underlying network topology, such as bias in particular connection patterns, weights can be easily assigned when computing the average interaction rank.

## Conclusion

Our contribution addresses the problem of large network reconstructions. The *Spectral Consensus Strategy* aims to reconstruct networks from high-dimensional dataset by overlapping subgraph parallel learning and consensus predictions. Although this approach is not intended to partition the data points, it takes advantage of spectral decomposition to identify tightly related vertices. We show by our experiments on both standard benchmark and real complex data that the performance of the proposed approach is extremely competitive. Our method is efficient from a computational viewpoint, its implementation is straightforward, and no effort has to be spent on hyper-parameter tuning.

## Additional file

Additional file 1 Contains complementary demonstrations as well as supplementary evaluations for the SCS approach. Specifically, Section 1 provides Propositions and associated *sketches of proof* that support our method. Complementary evaluations of the SCS first steps, namely SCS-*spectral* and SCS-*learn*, are given in Section 2. We also provide in Section 3 complementary evaluations of the SCS last step, named SCS-*consensus*. Execution time comparisons are given in Section 4. Supplementary statistics on the application to human gut microbiota close this Additional file 1. (PDF 606 kb)
