# Supplementary material for: Spectral consensus strategy for accurate reconstruction of large biological networks
Source: BMC Bioinformatics. 2016 Dec 13;17(Suppl 16):493. doi: 10.1186/s12859-016-1308-y (PMC5249011; doi:10.1186/s12859-016-1308-y)
Supplement: Additional file 1 — Contains complementary demonstrations as well as supplementary evaluations for the SCS approach. Specifically, Section 1 provides Propositions and associated sketches of proof that support our method. Complementary evaluations of the SCS first steps, namely SCS-spectral and SCS-learn, are given in Section 2. We also provide in Section 3 complementary evaluations of the SCS last step, named SCS-consensus. Execution time comparisons are given in Section 4. Supplementary statistics on the application to human gut microbiota close this Additional file 1. (PDF 606 kb) [file 12859_2016_1308_MOESM1_ESM.pdf]

Additional File 1

on the manuscript

Spectral Consensus Strategy for Accurate Reconstruction of Large Biological Networks

Séverine Affeldt Nataliya Sokolovska
Edi Prifti Jean-Daniel Zucker*

s.affeldt@ican-institute.org | nataliya.sokolovska@upmc.fr
| e.prifti@ican-institute.org | jean-daniel.zucker@ird.fr

*Contact author

1 Normalized Laplacian eigenvectors as path indicators

The null eigenvalues of the graph Laplacien matrices are associated with the number of *connected components* [1, 2]. Specifically, a subset of vertices $A_k \subset V$ is a connected component if (i) all intermediate points that lie on a path between two vertices of A_k also belong to A_k and (ii) there is no connection between the vertices of A_k and its complementary subset $\overline{A_k}$.

Proposition 1. *(Number of connected components and spectrum of L_{rw}). Let G be an undirected graph with non-negative weights. Then the multiplicity k of the eigenvalue 0 of L_{rw} equals the number of connected components $A_1, ...A_k$ in the graph. The eigenspace of 0 is spanned by the indicator vectors $\mathbb{1}_{A_i}$ of those components.*

For the case of finding $k > 2$ clusters, it can be shown that the k eigenvectors resulting from the spectral decomposition of the normalized Laplacian matrix L_{rw} correspond to the solution of the relaxed *NCut* minimization problem [3] (see main text, Methods)

The `SCS`-*spectral* phase of the `SCS` approach proposes to identify groups of vertices that are at a small *random walk* distance from each other within the graph $\mathcal{G}$ based on the element values of the Laplacian matrix eigenvectors. These subsets correspond to the best candidates for local subgraph reconstructions. In the following we assume that v_k is the k-th eigenvector of the normalized Laplacian matrix. We further assume that v_k is associated with the connected component A_k and that $v_k(i)$ is the ith element related to vertex i. In the ideal case, $i \in A_k \Rightarrow v_k(i) = 1$, otherwise $v_k(i) = 0$ [1].

Proposition 2. *(Similar eigenvector element values between connected vertices). Let $G = (V, E)$ be a weighted graph with connected components $\{A_k\}$. $\forall i \in A_k, \forall j \in V$ s.t. $v_k(j) > 0$, then $|v_k(i) - v_k(j)| = 0 \iff j$ is path connected with i.*

Sketch of Proof. The Laplacian eigenvalues λ_k can be computed by the Rayleigh quotient,

$$\lambda_k = \frac{v_k^T L_{rw} v_k}{v_k^T v_k} = \frac{\sum_{i \sim j} \left(v_k(i) - v_k(j)\right)^2}{\sum_i v_k(i)^2}. \tag{1}$$

Based on Proposition 1 and Equation 1, $(v_k(i) - v_k(j))^2 = 0$. Conversely, we assume that vertex j is not path connected with i. Then, as $i \in A_k$, the vertex j cannot belong to the connected component A_k. As the eigenvectors are indicators of the membership of each data points to the different communities, $(v_k(i) - v_k(j))^2 \neq 0$. Proposition 2 is given in the *ideal* case, which corresponds to well-separated components. Under small perturbations, it is expected that $v_k(i) \approx v_k(j)$ remains an indicator of a path between the two vertices i and j. ■

If $\mathcal{G}$ is a connected graph, the positive elements of the k-th eigenvector encode information about at most $(k-1)$ components.

Theorem 1. *(Fiedler's Nodal Domain). Let $G = (V, E, w)$ be a weighted connected graph, and let L be its Laplacian matrix. Let $0 = \lambda_1 < \lambda_2 \leq \cdots \leq \lambda_n$ be the eigenvalues of L and let $v_1, \ldots, v_n$ be the corresponding eigenvectors. For any $k \geq 2$, let $W_k = \{i \in V : v_k(i) \geq 0\}$. Then the graph induced by $\mathcal{G}$ on W_k has at most $k-1$ connected components.*

Thus, for a connected graph $\mathcal{G}$ and $k > 2$, one can expect to observe $|v_k(i) - v_k(j)| < \varepsilon$ while i and j belong to difference clusters A and B. Yet, according to [4], if there exists a subset of vertices S at a distance less than a *step* $\rho \geq 2$ from A that separates A and B, then v_k is such that

$$\begin{cases} \text{if } i \in A, \text{then } v_k(i) = 1, \\ \text{if } i \in B, \text{then } v_k(i) = -1, \\ \text{if } i \in S, \text{then } -1 + 2/\rho \leq v_k(i) \leq 1 - 2/\rho, \\ \text{if } i, j \text{ are adjacent vertices then } |v_k(i) - v_k(j)| \leq 2/\rho. \end{cases}$$

Taking $\rho = 2$ we obtain the case which is commonly used for separators. Hence, $|v_k(i) - v_k(j)|$ is a measure of the distance between the vertices i and j (*algebraic distance* [5]). This distance also reflects the *cluster assumption* indicating that close data points are expected to lie within the same cluster.

Finally, elements of greatest magnitude found in each of the first k eigenvectors are expected to be associated with variables involved in (nearly) connected components of $\mathcal{G}$.

Proposition 3. *(Dissimilar eigenvector element values between connected components). Let $G = (V, E)$ be a weighted graph with connected components $\{A_k\}$. $\forall (i, j) \in A_k, \forall l \in A_h, h \neq k, \min(|v_k(i) - v_k(l)|, |v_k(j) - v_k(l)|) > |v_k(i) - v_k(j)|$.*

Sketch of Proof. The first k eigenvectors of the normalised Laplacian matrix are indicators of the variable membership to each connected component. Furthermore, based on Proposition 2, one can establish that the first k eigenvectors are piecewise constant functions on their related connected components. ■

In summary, under ideal conditions, the first k eigenvectors of the normalized Laplacian matrix are the indicator vectors of the k connected components. In practice, the magnitude and sign of the eigenvector elements contain information on vertex membership *strength* to the corresponding component (Proposition 1, Appendix and main text, Methods). Furthermore, *path-connected* variables have similar eigenvector elements (Proposition 2.), that are distinct from the element of vertices belonging to a different component (Proposition 3.). Thus, subsets of nodes that correspond to large positive or negative eigenvector elements (retrieved in the `SCS`-*spectral* step) correspond to dense subgraphs (to be reconstructed in the `SCS`-*learn* step). These subgraphs associated to large eigenvector elements can be redundantly found in the first eigenvectors [6]. However, higher eigenvectors can also be used to identify different subsets of connected nodes, as observed in the context of anomalous graph detection [7].

2 Complementary evaluations of SCS-spectral & SCS-learn

The SCS approach embeds multiple reconstruction methods in a spectral framework to learn possibly oriented interactions from high-dimensional data by (i) combining the edges discovered from overlapping subgraphs (SCS-*spectral* & *learn* phases) and (ii) computing consensus predictions (SCS-*consensus* phase).

2.1 SCS-spectral & SCS-learn evaluations at different subgraph sizes

For these evaluations, two oriented benchmark networks have been considered: the ANDES benchmark network [8], composed of 223 nodes and 338 edges and the MUNIN benchmark network [9], composed of 1,041 nodes and 1,397 edges. Figure S6 provides the degree distribution of these networks. For ANDES, we randomly sampled 5 datasets of sizes 150 (Figure S1) and 200 (Figure S2) to perform the experiments under high-dimensional conditions, while we randomly sampled 5 datasets of sizes 935 for MUNIN (Figure S3).

The embedded reconstruction methods are 3off2 [10], a hybrid method that combines constraint-based and scoring approaches based on multivariate information measures (Figures S1-S3, top row), a hill-climbing algorithm using the Bayesian Dirichlet equivalent (BDe) score or the Bayesian Information Criteria (BIC) (Figures S1-S3, middle row) and ARACNE [11], a mutual information-based approach (Figures S1-S3, bottom row). We also considered a random classifier in our SCS-*spectral* and SCS-*learn* step evaluations (Figure S4).

Networks reconstructed in the SCS-*learn* phase based on the SCS-*spectral* subsets, are evaluated for different subgraph sizes (from 1.5% to 14% of vertices for the ANDES benchmark network [8], and from 5% to 11% of vertices for the MUNIN benchmark network [9]) and an increasing proportion of eigenvectors (up to 40%). Results are discussed in terms of *F-score* ($2 \times Prec \times Rec/(Prec + Rec)$). True positive edges that are falsely oriented are considered as false positive predictions (Figures S1-S3).

Alternative subset selections to the SCS-*spectral* phase were provided by spectral fuzzy C-means partitioning, spectral K-means clustering and recursive bi-partitioning (see main text). Overlapping subsets could be derived from the spectral fuzzy C-means partitioning. This alternative subset selection method performed generally better in our experiments than the other alternative approaches. Thus, we provide in this Appendix file comparisons of the SCS-*learn* step based on the SCS-*spectral* subsets with networks reconstructed from subsets provided by spectral fuzzy C-means partitioning (Figures S1-S3, middle column). Results obtained from random subset selection are also given as a mere comparison (Figures S1-S3, right column).

The association of the SCS-*spectral* and SCS-*learn* steps leads to higher *F-score* results as compared to reconstructions obtained with spectral fuzzy C-means partitioning. This improvement is achieved for a reasonable number of eigenvector and a large range of subgraph sizes, thus enabling a good trade-off between reconstruction quality and required number of subgraphs (Figures S1-S3).

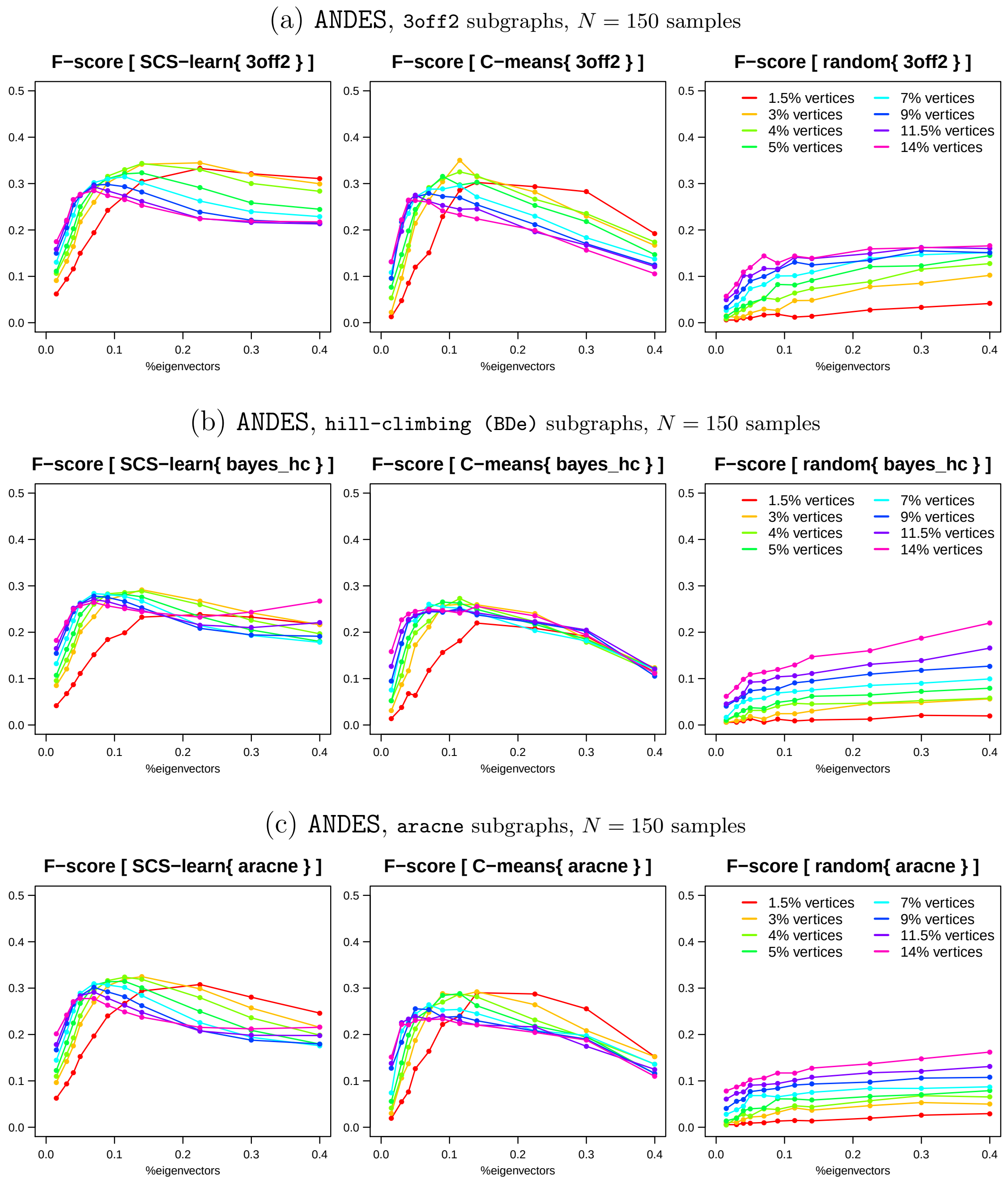

Figure S1: **SCS-learn evaluations for ANDES benchmark network** [223 nodes, 338 edges, $\langle k \rangle = 3.03$]. *F-score* results for an increasing proportion of eigenvectors (up to 40%) and different subgraph sizes (from 1.5% to 14% of vertices). Scores take misorientations into account. Each point is an average over 5 datasets of $n = 150$ samples. Three learning algorithms are embedded to reconstruct a network from subgraphs whose vertices are selected from the magnitude of eigenvector elements.

(a) ANDES, `3off2` subgraphs, $N = 200$ samples

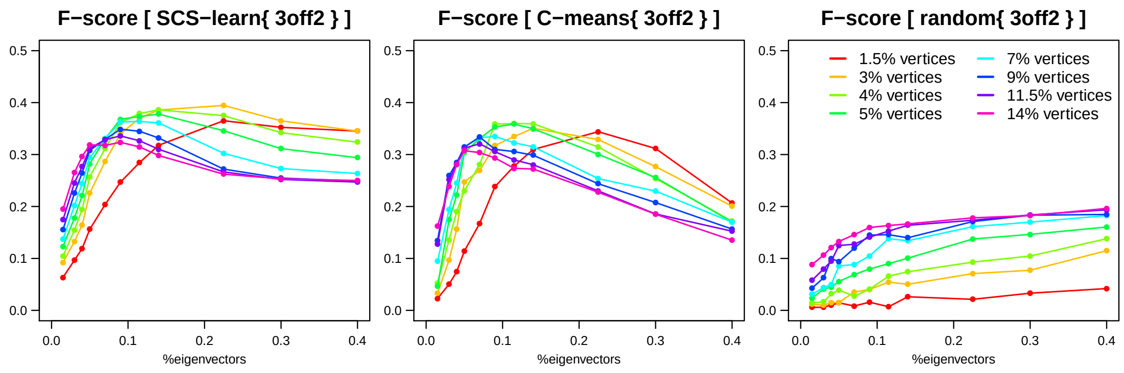

(b) ANDES, `hill-climbing (BDe)` subgraphs, $N = 200$ samples

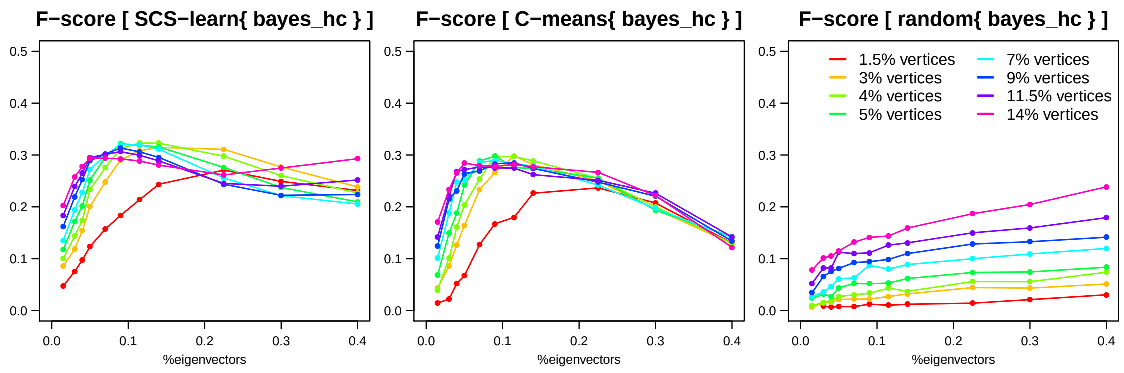

(c) ANDES, `aracne` subgraphs, $N = 200$ samples

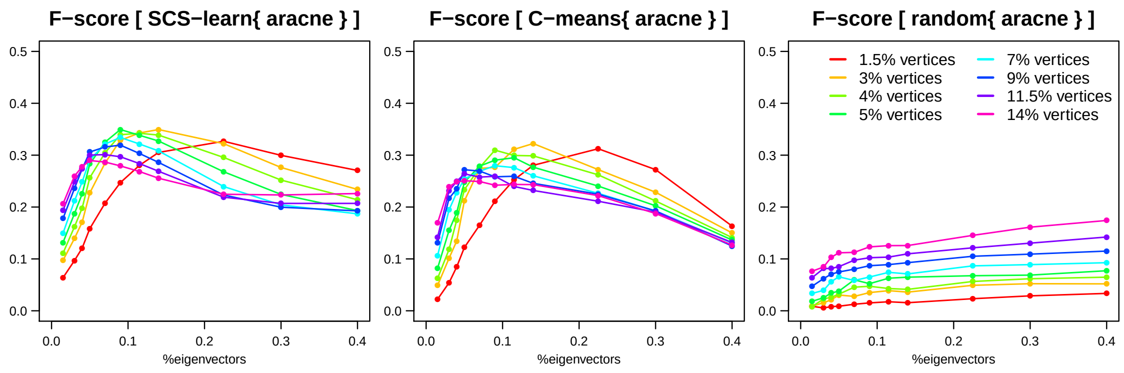

Figure S2: **SCS-learn evaluations for ANDES benchmark network** [223 nodes, 338 edges, $\langle k \rangle = 3.03$]. *F-score* results for an increasing proportion of eigenvectors (up to 40%) and different subgraph sizes (from 1.5% to 14% of vertices). Scores take misorientations into account. Each point is an average over 5 datasets of $n = 200$ samples. Three learning algorithms are embedded to reconstruct a network from subgraphs whose vertices are selected from the magnitude of eigenvector elements.

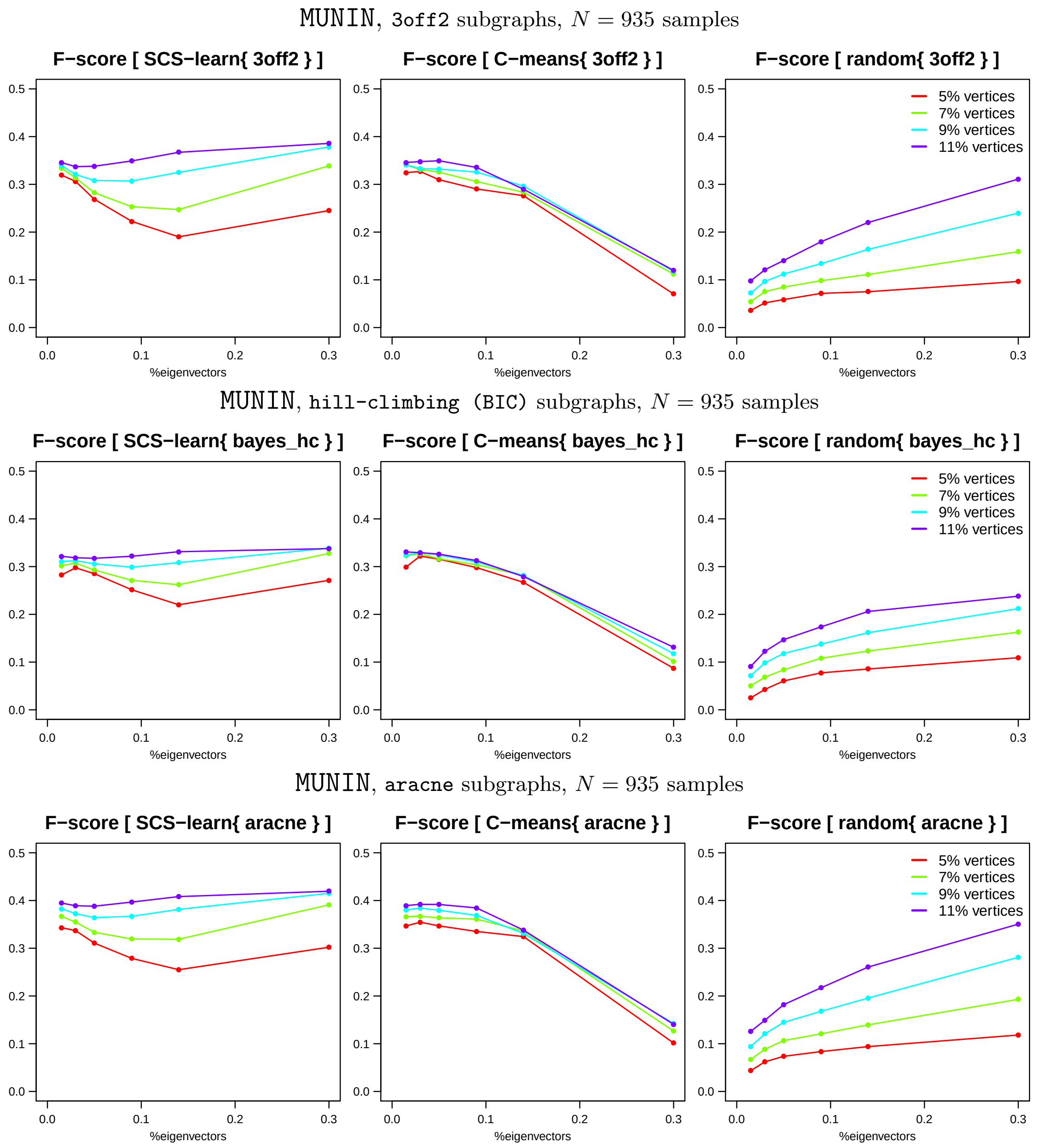

Figure S3: **SCS-learn evaluations for MUNIN benchmark network** [1,041 nodes, 1,397 edges, $\langle k \rangle = 2.68$]. *F-score* results for an increasing proportion of eigenvectors (up to 40%) and different subgraph sizes (from 5% to 11% of vertices). Scores take misorientations into account. Each point is an average over 5 datasets of $n = 935$ samples. Three learning algorithms are embedded to reconstruct a network from subgraphs whose vertices are selected from the magnitude of eigenvector elements.

2.2 Evaluations against random partition and random classifier

Networks reconstructed in the SCS-*learn* phase based on the SCS-*spectral* subsets, are compared to reconstruction obtained (i) with a random partitioning embedding `3off2`, `hc` or `aracne` methods (Figure S4, green solid line) and (ii) with the SCS-spectral subset identification embedding `3off2`, `hc`, `aracne` methods (Figure S4, red solid line) or a random classifier (Figure S4, orange solid line). Interestingly, the detection of connected subsets with the SCS-*spectral* step provide a very slight *Precision* improvement of the random classifier for the first few eigenvectors, showing that magnitude of the eigenvector elements provide information of relevant part of the underlying graph.

subgraphs of $m = 12$ variables (5%), $N = 150$ samples

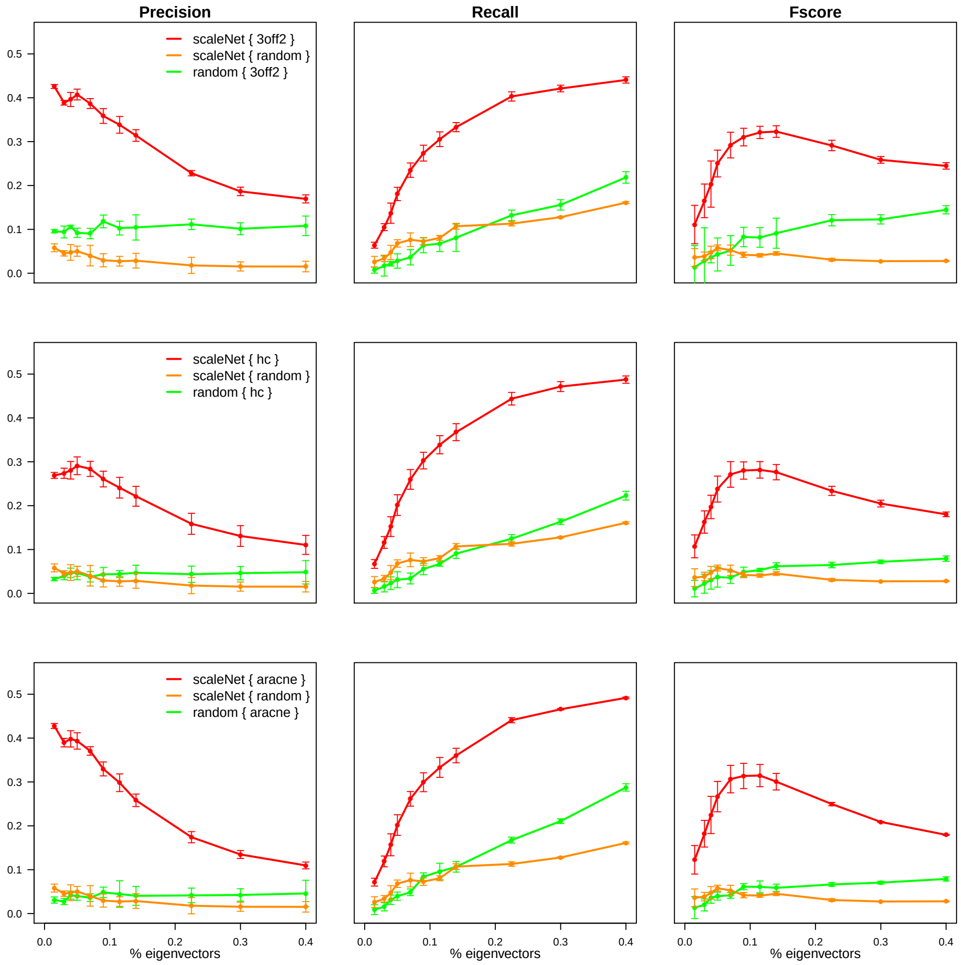

Figure S4: **SCS-learn evaluations for ANDES benchmark network against random partition and classifier** [223 nodes, 338 edges, $\langle k \rangle = 3.03$]. *F-score* results for an increasing proportion of eigenvectors (up to 40%) and subgraph sizes $m = 5\%$. Scores take misorientations into account. Each point is an average over 5 datasets of $n = 150$ samples. Three learning algorithms and a random classifier are embedded to reconstruct a network from subgraphs whose vertices are selected from the magnitude of eigenvector elements.

2.3 Coverage of the ANDES benchmark network with SCS-learn{3off2}

The `SCS`-*spectral* step retrieves subsets of vertices that are at a small *random walk* distance from each other based on eigenvector elements. As shown by the `SCS`-*learn Precision* improvement (Figure 2, main text; left column, red solid line), leading eigenvectors recover most of the relevant interactions. The *Recall* improvement (Figure 2, main text; middle column, red solid line) also shows that the remaining interactions are captured by non principal eigenvectors. Complementary statistics in Figure S5 show that 97% of the ANDES benchmark network can be recovered with 20,000 samples, and 62% with 150 samples, using 40% of the eigenvectors.

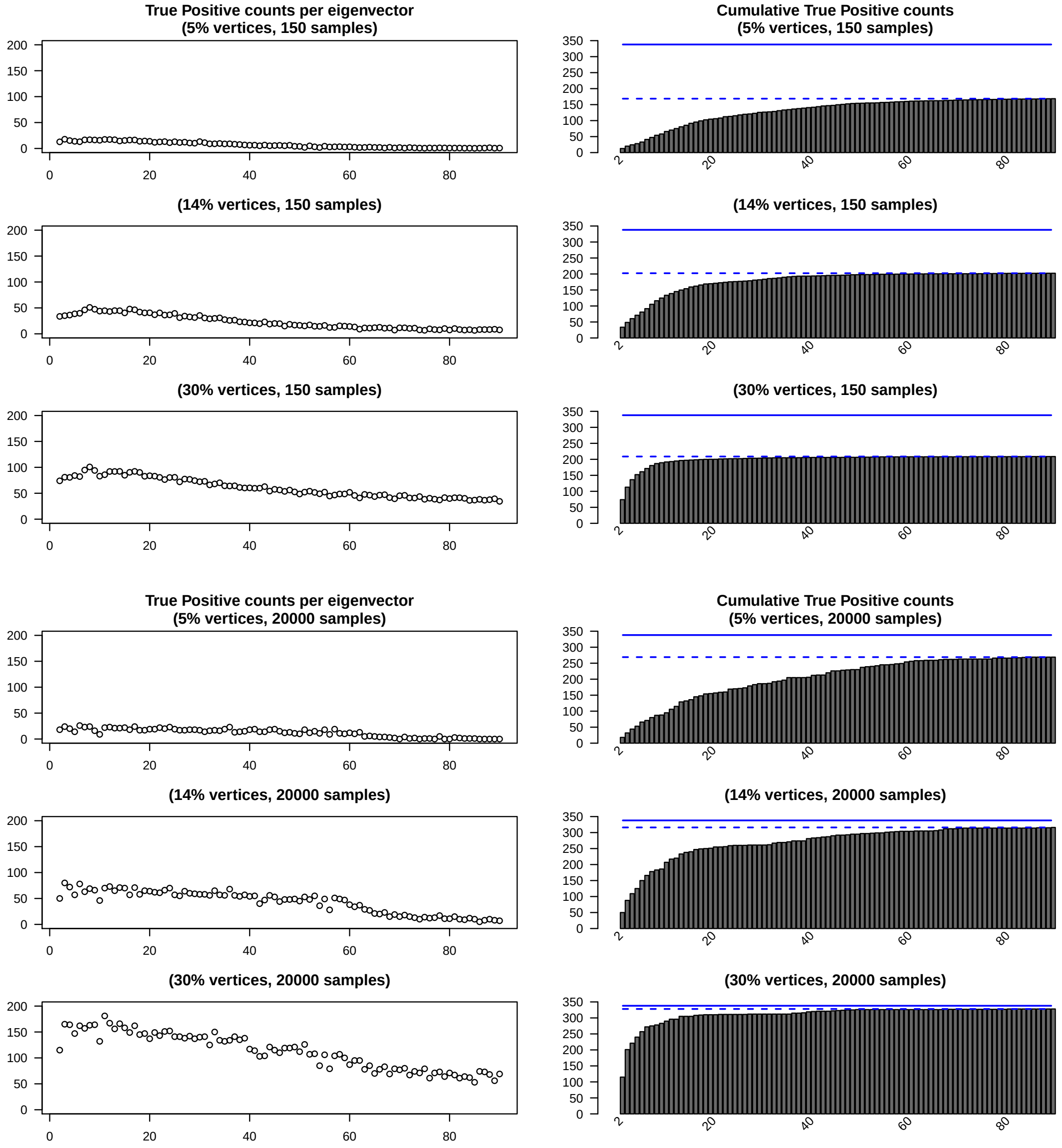

Figure S5: **Coverage of ANDES benchmark network with SCS-learn{3off2} at 150 and 20,000 samples** [ANDES: 223 nodes, 338 edges, $\langle k \rangle = 3.03$]. [left column] Count of true positive edges per eigenvector (up to 40%). [right column] Cumulative count of true positive edges as a function of eigenvector. The blue solid line indicates the number of interactions in the true network and the blue dashed line gives the maximum number of recovered true positive edges with `SCS`-*learn*{`3off2`}.

2.4 ANDES and MUNIN benchmark network degree distribution

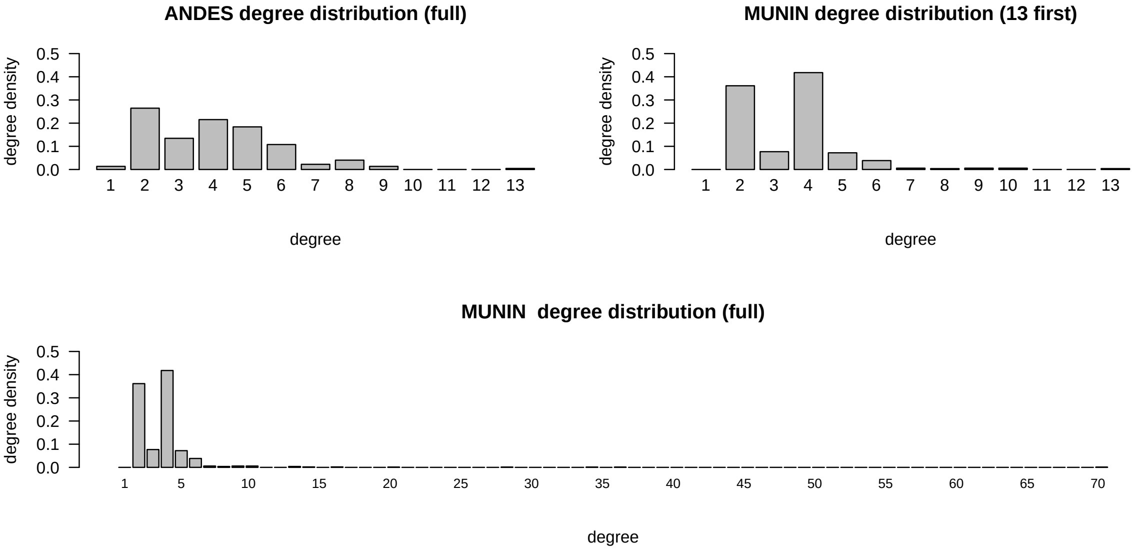

Figure S6: **Degree distribution comparison between ANDES and MUNIN benchmark networks** Comparison of degree distribution for ANDES [223 nodes, 338 edges, $\langle k \rangle = 3.03$, $k_{max} = 13$] and MUNIN [1,041 nodes, 1,397 edges, $\langle k \rangle = 2.68$, $k_{max} = 70$]. (top row) Full degree distribution of ANDES and partial degree distribution of MUNIN. (bottom row) Full degree distribution of MUNIN.

3 Complementary evaluations of the SCS-consensus phase

The benchmark networks used for the evaluation of the `SCS` consensus predictions are ANDES[8] (223 nodes, 338 edges, $\langle k \rangle = 3.03$) and MUNIN [9] (1,041 nodes, 1,397 edges, $\langle k \rangle = 2.68$). The degree distributions of these networks are given in Figure S6.

Evaluations on ANDES

Evaluations of consensus networks reconstructed from embedded learning approaches based on subgraphs of $m = 12, 16$ and 20 nodes, and using $n = 150$ and 200 samples are given in Figures S7-S8. The ANDES benchmark network is composed of 338 edges, thus scores for the consensus outcome are given based on the 338 first ranked edges $+/-25\%$ and 50%.

The consensus *Precision* scores (Figures S7-S8, left column, colored solid line) clearly outperform the individually embedded learning approaches (Figures S7-S8, left column, gray dashed lines) as the proportion of eigenvector grows.

All together, the `SCS`-*consensus* phase outcome provides high *F-score* network reconstructions (Figures S7-S8, right column, colored solid line) for a reasonable number of eigenvectors (proportion $\geq 11.5\%$). The `SCS`-*consensus* predictions also exhibit slightly higher *F-scores* when considering variable subsets of larger sizes in the `SCS`-*learn* phase (Figures S7-S8, top to bottom, colored solid line).

Evaluations on MUNIN

Evaluations of consensus networks reconstructed from embedded learning approaches based on subgraphs of $m = 16, 32$ and 53 nodes, and using $n = 700$ and 935 samples are given in Figure S9. The MUNIN benchmark network is composed of 1,397 edges, thus scores for the consensus outcome are given based on the 1,397 first ranked edges $+/-25\%$ and 50%.

The consensus *Precision* scores (Figure S9, left column, colored solid line) clearly outperform the individually embedded learning approaches (Figure S9, left column, gray dashed lines) as the proportion of eigenvector grows.

All together, the `SCS`-*consensus* phase outcome provides relatively high *F-score* network reconstructions (Figure S9, right column, colored solid line) for a reasonable number of eigenvectors. The `SCS`-*consensus* predictions also exhibit slightly higher *F-scores* when considering variable subsets of larger sizes in the `SCS`-*learn* phase (Figure S9, top to bottom, colored solid line).

(a) ANDES, $\mathbf{n} = \mathbf{150}$ **samples**, subgraphs of $\mathbf{m} = \mathbf{12}$ variables (5%)

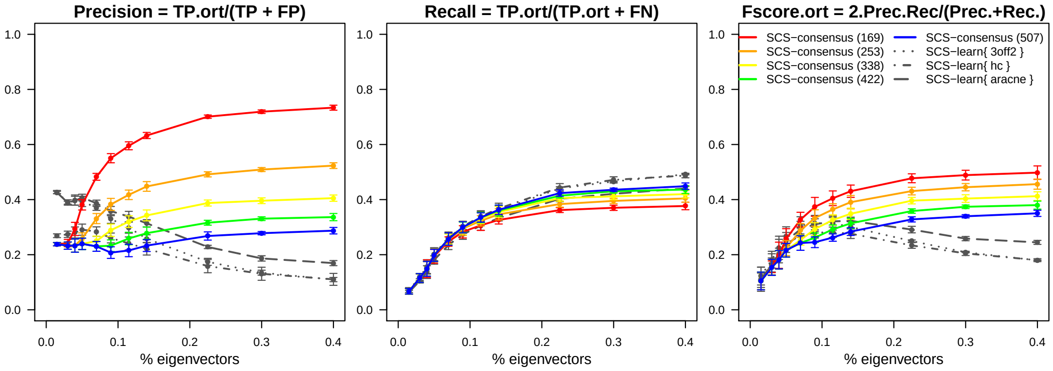

(b) ANDES, $\mathbf{n} = \mathbf{150}$ **samples**, subgraphs of $\mathbf{m} = \mathbf{16}$ variables (7%)

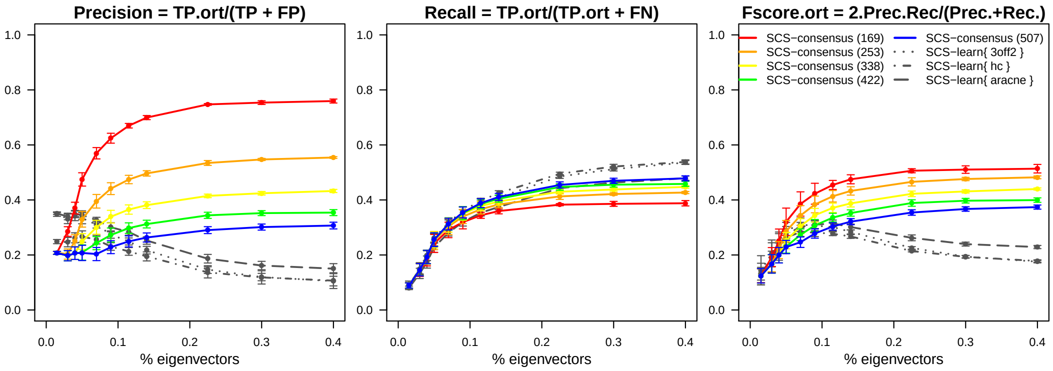

(c) ANDES, $\mathbf{n} = \mathbf{150}$ **samples**, subgraphs of $\mathbf{m} = \mathbf{21}$ variables (9%)

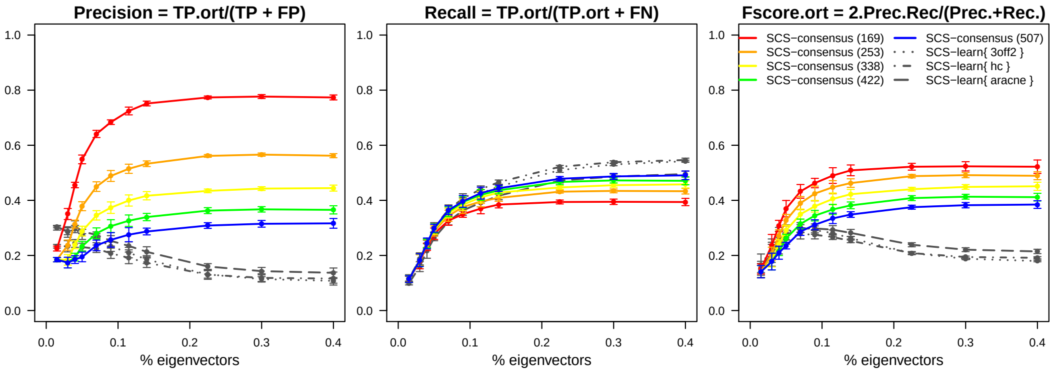

Figure S7: **SCS-consensus evaluations of ANDES network reconstruction** [$n = 150$, $m = 12, 16, 21$]. *Precision*, *Recall* and *F-score* results for the oriented ANDES network (223 nodes, 338 edges, $\langle k \rangle = 3.03$). Results are given for an increasing proportion of eigenvectors (horizontal axis) and subgraphs of 12, 16 and 21 nodes. Each point is an average over 5 datasets of size $n = 150$ samples. Scores take misorientations into account (see main text). The individual reconstructions (gray dashed lines) are combined in a consensus network (colored solid lines). Scores are computed based on a range of top consensus edges which corresponds to the total number of ANDES edges +/− 25% or 50%.

(a) ANDES, $\mathbf{n = 200}$ **samples**, subgraphs of $\mathbf{m = 12}$ variables (5%)

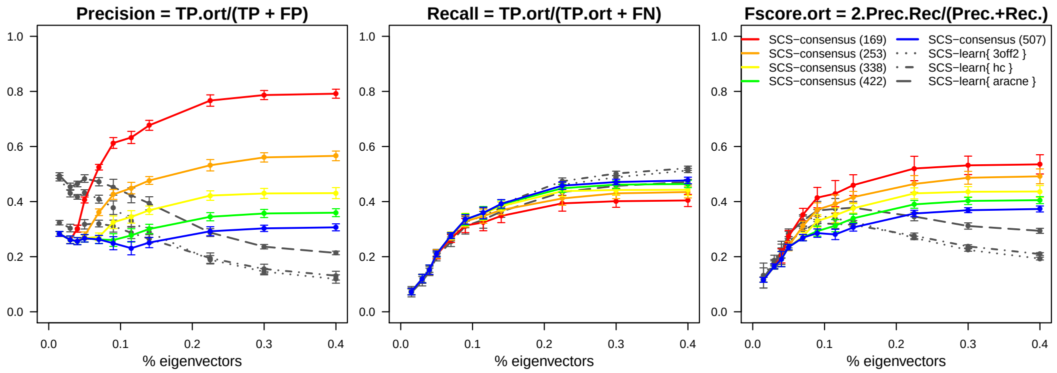

(b) ANDES, $\mathbf{n = 200}$ **samples**, subgraphs of $\mathbf{m = 16}$ variables (7%)

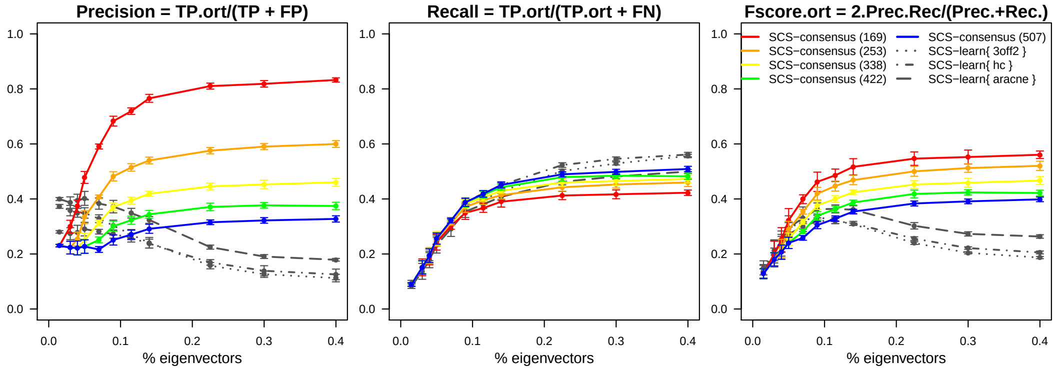

(c) ANDES, $\mathbf{n = 200}$ **samples**, subgraphs of $\mathbf{m = 21}$ variables (9%)

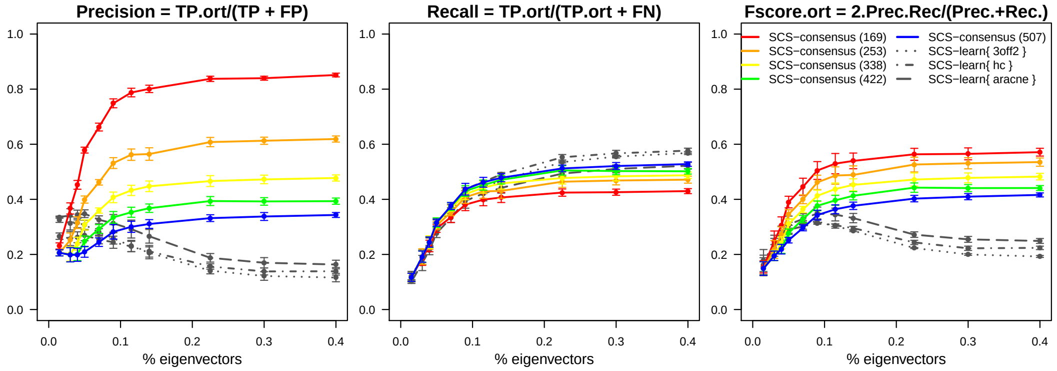

Figure S8: **SCS-consensus evaluations of ANDES network reconstruction** [$n = 200$, $m = 12, 16, 21$]. *Precision*, *Recall* and *F-score* results for the oriented ANDES network (223 nodes, 338 edges, $\langle k \rangle = 3.03$). Results are given for an increasing proportion of eigenvectors (horizontal axis) and subgraphs of 12, 16 and 21 nodes. Each point is an average over 5 datasets of size $n = 200$ samples. Scores take misorientations into account (see main text). The individual reconstructions (gray dashed lines) are combined in a consensus network (colored solid lines). Scores are computed based on a range of top consensus edges which corresponds to the total number of ANDES edges +/− 25% or 50%.

(a) MUNIN, $\mathbf{n = 935}$ **samples**, subgraphs of $\mathbf{m = 32}$ variables (3%)

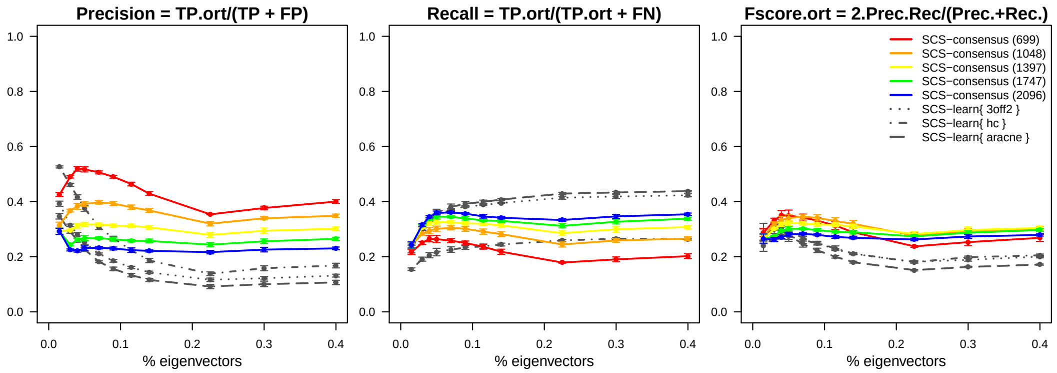

(b) MUNIN, $\mathbf{n = 935}$ **samples**, subgraphs of $\mathbf{m = 53}$ variables (5%)

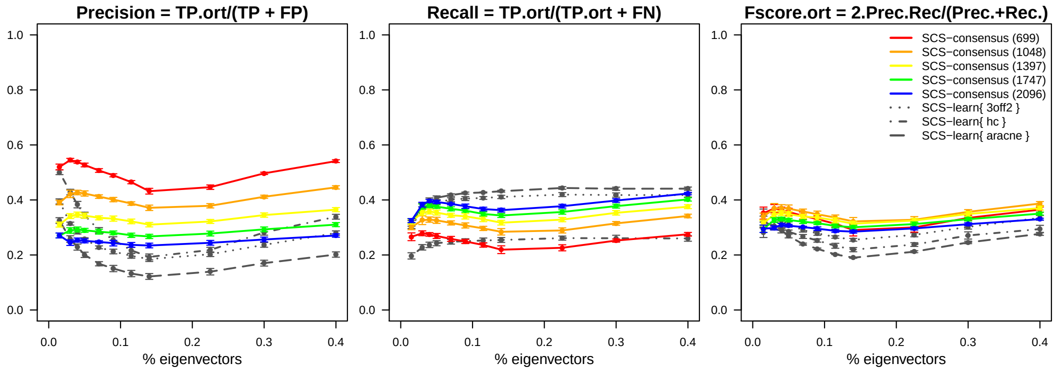

(c) MUNIN, $\mathbf{n = 935}$ **samples**, subgraphs of $\mathbf{m = 73}$ variables (7%)

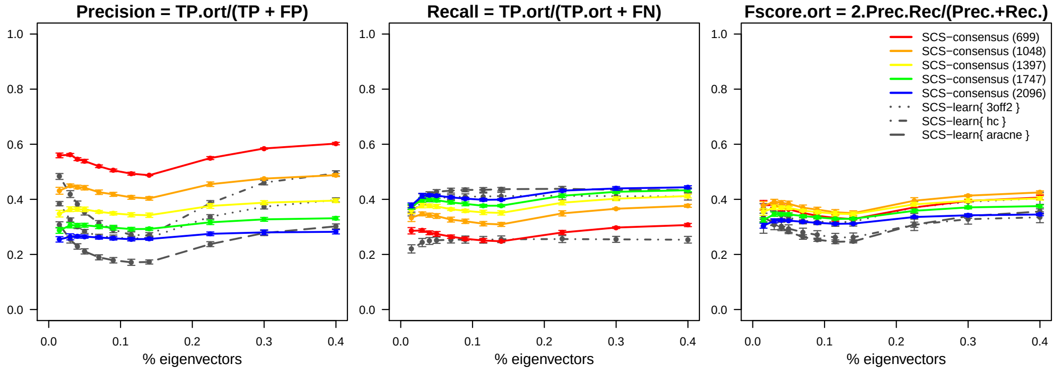

Figure S9: **SCS-consensus evaluations of MUNIN network reconstruction** [$n = 935$, $m = 32, 53, 73$]. *Precision*, *Recall* and *F-score* results for the oriented MUNIN network (1041 nodes, 1397 edges, $\langle k \rangle = 2.68$). Results are given for an increasing proportion of eigenvectors (horizontal axis) and subgraphs of 32, 53 and 73 nodes. Each point is an average over 5 datasets of size $n = 935$ samples. Scores take misorientations into account (see main text). The individual reconstructions (gray dashed lines) are combined in a consensus network (colored solid lines). Scores are computed based on a range of top consensus edges which corresponds to the total number of MUNIN edges +/− 25% or 50%.

4 Execution time comparisons

The SCS approach embeds multiple reconstruction methods in a spectral framework to learn possibly oriented interactions from high-dimensional data by (i) combining the edges discovered from overlapping subgraphs (main text, Figure 1, SCS-*spectral* & *learn*, (a-b)) and (ii) computing a consensus network (main text, Figure 1, SCS-*consensus*, (c)).

We provide in this supplementary file execution times in seconds for the different phases and sub-steps of these phases, namely:

(a) SCS-*spectral*

- $\succ$ W : mutual information matrix computation
- $\succ$ L : normalized Laplacian matrix computation
- $\succ$ $\{v_k, \lambda_k\}$: normalized Laplacian matrix decomposition

(b) SCS-*learn*

- $\succ$ $\{\mathcal{G}_{v_k,l}^{m,+/-}\}$: local reconstructions of subgraphs
  (subgraphs from the m most *positive* and m most *negative* elements of v_k)
- $\succ$ $\mathcal{G}_l$ embedded reconstruction
  (combination of subgraphs for an individual reconstruction method)

(c) SCS-*consensus*

- $\succ$ $\{\{\mathcal{G}_{v_k,l}^{m,+/-}\}\}_L$: local reconstructions of subgraphs for L individual methods
- $\succ$ $\{\mathcal{G}_l\}_L$: embedded reconstruction for L individual methods
- $\succ$ $\mathcal{G}$ consensus reconstruction

In the following, we consider execution times for the reconstruction of human gut microbial ecosystem from a complex biological dataset generated by high-throughput sequencing. This dataset involves p = 2,101 CAGs (co-abundant gene groups, see main text) and n = 663 samples from patients recruited in the MetaHit project [12].

Table S1 gives the execution times for the reconstruction of the gut microbial ecosystem using the SCS-*spectral* & SCS-*learn* frameworks. Table S2 provides the overall times for the complete SCS reconstruction. The sufficient proportion of eigenvectors for these SCS reconstructions was evaluated at 3% (63 eigenvectors). The proportion of variables in each subgraph has been set to 1.9% (40 variables) to guarantee non high-dimensional conditions. Lastly, execution times for each reconstruction method outside of any SCS framework are given in Table S3.

	W	L	$\{v_k, \lambda_k\}$	$\{\{\mathcal{G}^{m,\bullet}_{v_k,l}\}\}_L$	$\mathcal{G}_l$	**Total (s.)**
3off2				11	1,161	3,078
ARACNE	1880	13	13	12	595	2,513
hill-climbing				17	396	2,319

Table S1: SCS-*spectral* & *learn* execution times in seconds [2,101 variables, 663 samples]

	W	L	$\{v_k, \lambda_k\}$	$\{\{\mathcal{G}^{m,\bullet}_{v_k,l}\}\}_L$	$\{\mathcal{G}_l\}_L$	$\mathcal{G}$	**Total (s.)**
consensus	1880	13	13	17	1,161	52	3,136

Table S2: SCS-*consensus* execution time in seconds when integrating 3off2, ARACNE and hill-climbing [2,101 variables, 663 samples]

**original method**	**Total (s.)**
3off2	30,531
ARACNE	1,996
hill-climbing	no convergence in 48 hours

Table S3: Original reconstruction method execution times in seconds [2,101 variables, 663 samples]

The SCS method could reconstruct the gut microbial ecosystem in 3,136 sec., while the hill-climbing algorithm did not converge in 48 hours. The ARACNE method allows a faster reconstruction (1,996 sec.) than the SCS, the 3off2 or the hill-climbing methods. Nevertheless, the SCS framework provides a more accurate reconstruction within reasonable time (3,136 sec.).

All together, our experiments on both standard benchmark and real complex data demonstrate that our SCS approach is efficient and accurate under high-dimensional settings, addressing a major issue in the field. A package is currently being implemented to provide the community with this novel robust reconstruction method.

5 SCS network enrichment in species sharing same assembly contigs

We compared the SCS results with the pairwise network of 307 edges reconstructed by Nielsen *et al* [12]. Specifically, we considered common predictions between the SCS top-ranked edges (*%edges* in $\{2\%, 100\%\}$) and Nielsen *et al*'s pairwise network (# and *%Common edges*). For these common interaction subsets, we evaluated the enrichment in edges that relate MGS with genomic elements sharing the same assembly, as this brings strong biological evidence for such predicted relationships (Table S4 and Figure S10).

SCS network		SCS and pairwise networks			
%edges	#edges	#Common edges	%Common edges	%Common edges, same orientation	SCS edges, **contig enrichment** (χ^2)
2%	128	55	18%	75%	$\mathbf{2.23\times10^{-6}}$
3%	192	76	25%	74%	$\mathbf{4.45\times10^{-9}}$
5%	319	113	37%	78%	$\mathbf{3.84\times10^{-10}}$
10%	639	124	40%	79%	$\mathbf{2.15\times10^{-8}}$
15%	958	126	41%	79%	$\mathbf{1.04\times10^{-8}}$
20%	1278	129	42%	78%	$\mathbf{3.37\times10^{-9}}$
25%	1597	131	43%	78%	$\mathbf{1.55\times10^{-9}}$
50%	3194	145	47%	77%	$\mathbf{3.56\times10^{-9}}$
100%	6389	146	48%	76%	$\mathbf{2.38\times10^{-9}}$

Table S4: **SCS predictions enrichment in edges between MGS with genomic elements sharing the same assembly contigs, χ^2 test**. Comparisons between the SCS and pairwise interactions [12]. *Common edges* are considered for an increasing number of SCS edges. *%Common edges, same orientation* column indicates the number of edges common to the SCS and Nielsen *et al.* networks with identical orientation. SCS *edges, contig enrichment (χ^2)* column indicates the enrichment in edges between MGS with genomic elements sharing the same assembly contigs.

Interestingly, although not all edges are expected to have *genome assembly* associations, the SCS predicted edges are significantly enriched in interactions related to such assembly associations compare to the pairwise network inferred by Fisher's exact test. Furthermore, the interactions with *genome assembly* associations are found in the top-ranked edges predicted by SCS, as can be seen from Figure S10.

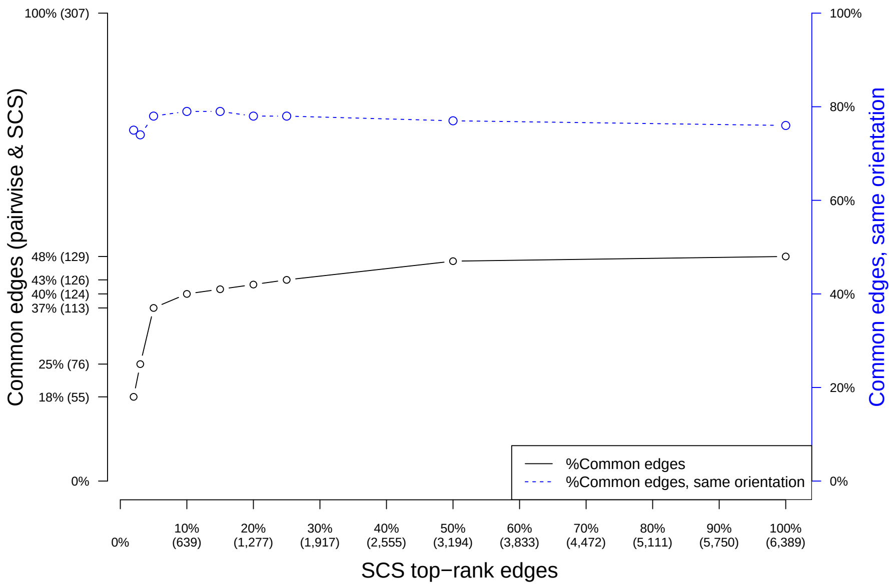

Figure S10: **SCS and pairwise common predictions**.

References

[1] Luxburg, U.: A tutorial on spectral clustering. Statistics and Computing **17**(4), 395–416 (2007)

[2] Mohar, B., Alavi, Y., Chartrand, G., Oellermann, O.: The laplacian spectrum of graphs. Graph theory, combinatorics, and applications **2**(871-898), 12 (1991)

[3] Shi, J., Malik, J.: Normalized cuts and image segmentation. IEEE Transactions on Pattern Analysis and Machine Intelligence **22**(8), 888–905 (2000)

[4] Pothen, A., Simon, H.D., Liu, K.-P.P.: Partitioning sparse matrices with eigenvectors of graphs. Technical report, NASA Ames Research Center (1989)

[5] Fiedler, M.: Algebraic connectivity of graphs. Czechoslovak Mathematical Journal **23**(98) (1973)

[6] Kleinberg, J.M.: Authoritative sources in a hyperlinked environment. Journal of the ACM (JACM) **46**(5), 604–632 (1999)

[7] Miller, B., Bliss, N., Wolfe, P.J.: Subgraph detection using eigenvector l1 norms. In: Advances in Neural Information Processing Systems, pp. 1633–1641 (2010)

[8] Conati, C., Gertner, A.S., VanLehn, K., Druzdzel, M.J.: On-line student modeling for coached problem solving using bayesian networks. In: User Modeling, pp. 231–242 (1997). Springer

[9] Andreassen, S., Jensen, F., Andersen, S., Falck, B., Kjærulff, U., Woldbye, M., Sørensen, A., Rosenfalck, A., Jensen, F.: Muninan expert emg assistant. Computer-aided electromyography and expert systems **21** (1989)

[10] Affeldt, S., Verny, L., Isambert, H.: 3off2: A network reconstruction algorithm based on 2-point and 3-point information statistics. BMC Bioinformatics **17**(S-2), 12 (2016)

[11] Margolin, A.A., Nemenman, I., Basso, K., Wiggins, C., Stolovitzky, G., Favera, R.D., Califano, A.: Aracne: An algorithm for the reconstruction of gene regulatory networks in a mammalian cellular context. BMC Bioinformatics **7**(Suppl 1) (2006)

[12] Nielsen, H.B., Almeida, M., Juncker, A.S., Rasmussen, S., Li, J., Sunagawa, S., Plichta, D.R., Gautier, L., Pedersen, A.G., Le Chatelier, E., *et al.*: Identification and assembly of genomes and genetic elements in complex metagenomic samples without using reference genomes. Nature biotechnology **32**(8), 822–828 (2014)
